# Does stochastic resonance improve performance for individuals with higher autism-spectrum quotient?

**DOI:** 10.3389/fnins.2023.1110714

**Published:** 2023-04-14

**Authors:** Pratik Raul, Kate McNally, Lawrence M. Ward, Jeroen J. A. van Boxtel

**Affiliations:** ^1^Discipline of Psychology, Faculty of Health, University of Canberra, Canberra, ACT, Australia; ^2^Department of Psychology, University of British Columbia, Vancouver, BC, Canada; ^3^Djavad Mowafaghian Centre for Brain Health, University of British Columbia, Vancouver, BC, Canada; ^4^Turner Institute for Brain and Mental Health, School of Psychological Sciences, Monash University, Melbourne, VIC, Australia

**Keywords:** stochastic resonance, autism-spectrum disorders, visual noise, neural noise, visual identification, enhanced performance

## Abstract

While noise is generally believed to impair performance, the detection of weak stimuli can sometimes be enhanced by introducing optimum noise levels. This phenomenon is termed ‘Stochastic Resonance’ (SR). Past evidence suggests that autistic individuals exhibit higher neural noise than neurotypical individuals. It has been proposed that the enhanced performance in Autism Spectrum Disorder (ASD) on some tasks could be due to SR. Here we present a computational model, lab-based, and online visual identification experiments to find corroborating evidence for this hypothesis in individuals without a formal ASD diagnosis. Our modeling predicts that artificially increasing noise results in SR for individuals with low internal noise (e.g., neurotypical), however not for those with higher internal noise (e.g., autistic, or neurotypical individuals with higher autistic traits). It also predicts that at low stimulus noise, individuals with higher internal noise outperform those with lower internal noise. We tested these predictions using visual identification tasks among participants from the general population with autistic traits measured by the Autism-Spectrum Quotient (AQ). While all participants showed SR in the lab-based experiment, this did not support our model strongly. In the online experiment, significant SR was not found, however participants with higher AQ scores outperformed those with lower AQ scores at low stimulus noise levels, which is consistent with our modeling. In conclusion, our study is the first to investigate the link between SR and superior performance by those with ASD-related traits, and reports limited evidence to support the high neural noise/SR hypothesis.

## 1. Introduction

Noise, defined here as unwanted variability, is generally considered to be a nuisance that disrupts information processing (McDonnell and Abbott, 2009). Indeed, when noise is added to the perceptual system, performance generally decreases (Mackworth, 1965). However, in certain circumstances, noise can be beneficial for a system (McDonnell and Abbott, 2009). This effect has been extensively studied in engineering, but there are examples in biological systems as well, which includes postural stability (Costa et al., 2007; Kaut et al., 2016), perceptual accuracy (Piana et al., 2000), and visual information processing in the visually impaired (Itzcovich et al., 2017). The phenomenon where noise increases performance (or other outcome measures) is called ‘stochastic resonance’ (SR) (Wiesenfeld and Moss, 1995; Ward et al., 2002; Moss et al., 2004; McDonnell and Abbott, 2009). SR is characterized by an inverted U-shaped function where performance is gradually increased by the addition of noise until it reaches peak performance, after which further addition of noise results in the gradual decline of performance (McDonnell and Abbott, 2009). While SR has been mostly investigated in engineering, it is now being researched across medical science and neuroscience, because of its capability to enhance or improve sensory, motor, and physiological functions.

Although adding noise to improve performance has large potential impacts, adding noise does not help in most tasks, because SR is mostly limited to sub-threshold stimuli. Adding noise to supra-threshold tasks typically will not be beneficial, although there are examples of supra-threshold SR (McDonnell et al., 2006). Moreover, even when SR is found on average, adding noise does not produce a benefit in everyone (Aihara et al., 2008, 2010). This suggests that apart from external factors (e.g., amount of noise in the stimulus, i.e., external noise), the appearance of SR also depends on internal factors specific to an individual. Aihara et al. (2008) suggested that one such internal factor is the level of noise in the brain itself (i.e., neural noise), termed ‘internal noise’. The results by Aihara et al. (2010) suggested that that SR will fail to occur when internal noise is high in an individual. In other words, adding external noise to a system (such as the brain) that already has high amounts of internal noise will result in being overwhelmed with too much noise. On the other hand, adding external noise to a system with low internal noise levels can result in SR, because noise can push a sub-threshold signal above threshold. The variability in internal noise could explain why SR is generally not observed consistently across all populations.

A population suspected to have high levels of internal noise is individuals diagnosed with Autism Spectrum Disorder (ASD) (Rubenstein and Merzenich, 2003). ASD is a neurodevelopmental disorder that is characterized by restrictive and repetitive behaviors, and social communication deficits (American Psychiatric Association, 2013). It is hypothesized that behavioral characteristics observed in ASD can be linked to high internal noise or neural variability in the autistic brain^1^ (Rubenstein and Merzenich, 2003; Simmons et al., 2009; Milne, 2011). This high internal noise in ASD is linked to an imbalance in excitation/inhibition ratios in the brain (Rubenstein and Merzenich, 2003). It is believed that the high neural noise in ASD results in unreliable and less predictable representations of the environment (Dinstein et al., 2015). This hypothesis is supported by imaging and electroencephalogram (EEG) studies [however, there are some exceptions (Coskun et al., 2009; Butler et al., 2017)]. For instance, an EEG study showed that an autistic group displayed higher *intra*-participant variability (trial-by-trial variability) when compared to the typically developing group (TD) (Milne, 2011). Further, an fMRI study showed increased trial-by-trial variability in BOLD (blood oxygen level dependent) responses within visual, auditory, and somatosensory cortices in autistic adults when compared to neurotypical adults (Dinstein et al., 2012). The increased trial-by-trial variability in neural activity in ASD was also discussed in a review by David et al. (2016). Moreover, a recent study also found high *inter*-individual variability in ASD, and estimates of internal noise were significantly correlated with the severity of ASD symptoms [measured using the Autism Diagnostic Observation Schedule or ADOS (Park et al., 2017)]. However, there are also some studies that have not found a significant relationship between internal noise and ASD (Manning et al., 2015). Recently, Manning et al. (2017) also showed that internal noise levels were not significantly different in the autism group (although they were higher). Overall, there is good, but equivocal, evidence indicating that higher-than-typical neural noise may be a characteristic of ASD.

This pattern of a higher internal noise level is not confined to individuals diagnosed with ASD. It is also reported in typically developing individuals (TD) who exhibit a high level of autistic traits. The degree to which an individual exhibits autistic traits can be measured using the Autism-spectrum Quotient (AQ), introduced by Baron-Cohen et al. (2001), or the Social Responsiveness Scale (SRS-2) (Constantino and Gruber, 2012). Vilidaite et al. (2017) showed, using a psychophysical double-pass paradigm, that the amount of internal noise was positively correlated with the degree to which participants exhibited autistic traits on the AQ [see also (Orchard et al., 2021)].

The higher levels of internal noise in ASD provide a convenient explanation of decreased performance on various tasks such as in contour integration tasks (Mihaylova et al., 2021), second-order (or complex) detection tasks (Bertone et al., 2005), and orientational discrimination tasks (Park et al., 2017).

However, interestingly, autistic individuals also show superior performance on several tasks. There are well-documented examples of savant capabilities in ASD (Howlin et al., 2009), and it is also well-reported that autistic individuals, on average, show enhanced performance on some perceptual tasks such as visual search, and block design tasks (Mottron and Burack, 2001; Mottron et al., 2006). In an effort to parsimoniously explain both enhanced and reduced perceptual capabilities in ASD in light of the increased neural noise in that population, Simmons et al. (2009) suggested that the increase in performance (e.g., detection ability) could be due to SR. There is some circumstantial evidence for this hypothesis. For instance, one study showed that children with ASD displayed a small performance enhancement in a visuo-spatial static task for first order contrast detection (luminance-defined) in noise, whereas in the same task, performance declined for second order contrast detection (texture-defined) (Bertone et al., 2005). Simmons et al. (2009) suggested that the increase in performance (detectability) in the first order luminance-defined gratings in noise was due to an increase in internal noise in their visual system, subsequently enlarging the signal moderately through SR. However, for the second order task, this SR effect does not occur, as extracting information from the second order task requires additional processing (such as integrating information across more visual filters) and is thus nosier by itself (Schofield and Georgeson, 1999; Simmons et al., 2009). Together with the increased noisy stimulus, the overall level of noise may have overwhelmed the visual system and hence, a decline in performance occurred.

Indeed, it appears that most tasks where increased performance in ASD is found are in visually “simple” tasks such as visual search tasks (Kaldy et al., 2016), while many of the more complex counterparts show decreased performances in ASD (Bertone et al., 2005).

There has been no direct investigation into whether SR arising from higher levels of internal noise is the cause for the increased performance in ASD on some tasks. Here, we will investigate whether higher trait levels on the Autism Quotient correlated with increased performance through SR. We performed this research in the typically developing (TD) population, because this gives us an opportunity to (1) have a larger sample size, and (2) allows us to study and model how noise could affect individuals within the broader population, potentially increasing generalizability. If the hypothesis proposed by Simmons et al. (2009) is true, then performance in conditions when external noise is low or zero should be better for individuals with higher levels of AQ (and presumably higher levels of internal noise) compared to that of individuals with lower AQ scores (and presumably lower levels of internal noise).

## 2. Modeling stochastic resonance in M-alternative choice tasks

To substantiate this prediction (and others) further, we constructed a model to explain detection performance under noisy conditions in an M-alternative choice task. M-alternative choice tasks have been modeled in the past (Tanner and Swets, 1954; Swets et al., 1961; Hacker and Ratcliff, 1979; Green and Dai, 1991; Wickens, 2002; Macmillan and Creelman, 2005; DeCarlo, 2012). Here we extend this to conditions where the signal strength is sub-threshold, and is only correctly identified due to the presence of noise (i.e., SR).

The proportion correct, Pc, in an M-alternative choice task is in general


(1) $$Pc=\overset{\infty }{\underset{-\infty }{\int }}{\left[\Phi \left(x\right)\right]}^{M-1}\;\phi \left(x-s\right)dx,$$

where ϕ is the cumulative normal density function, ϕ is the normal probability density function, and *s* is the signal strength, and M is the number of alternatives (including the target). Here, ϕ describes the chance of a certain signal value occurring given an average signal strength (*s*), and noise i.e., the standard deviation, σ, of ϕ ; here set at (1). $\phi \left(x\right)$ describes the chance of the *M*-1 non-target alternatives being smaller than that (noisy) signal value. Equation (1) describes the overall probability that the target signal is the largest value, i.e., proportion correct, or accuracy in ideal cases.

To model stochastic resonance, we include a threshold, τ, and assume that the signal strength is below threshold, i.e., *s* < τ. The approach is similar to above, but one of the alternatives will only be chosen when it is both the largest, and also above threshold. When none of the alternatives is above threshold, an unbiased guess will result. Therefore, there are two situations that can lead to a correct response. First, the signal value (*x*) is above the threshold, and it is also the largest:


(2) $$Pc_{s\geq \tau }=\overset{\infty }{\underset{\tau }{\int }}{\left[\Phi \left(x\right)\right]}^{M-1}\;\phi \left(x-s\right)dx,$$

which is similar to Equation 1, except that the integral starts from τ, to ensure that the signal is above threshold.

Second, all M alternatives are below threshold, and the correct stimulus is chosen by chance


(3) $$Pc_{s<\tau }=\frac{1}{M}{\left[\Phi \left(\tau \right)\right]}^{M-1}\;\Phi \left(\tau -s\right),$$

where $\phi \left(\tau -s\right)$ represents the probability of the signal stimulus being below threshold, and ${\left[\Phi \left(\tau \right)\right]}^{M-1}$ represents the probability that all other *M*-1 alternatives are also below threshold. The probability of then choosing the correct stimulus is 1/*M*. The overall percentage correct is then the sum of *Pc*_s **≥** τ_ and *Pc*_s < τ_


(4) $$Pc=\overset{\infty }{\underset{\tau }{\int }}{\left[\Phi \left(x\right)\right]}^{M-1}\;\phi \left(x-s\right)dx+\frac{1}{M}{\left[\Phi \left(\tau \right)\right]}^{M-1}\;\Phi \left(\tau -s\right).$$

Next, we include both contributions of internal noise (σ_int_) and external noise (σ_ext_). Therefore, we explicitly model the standard deviation (σ) of the signal and alternative distributions (which we assume to be the same), yielding


(5) $$\begin{array}{l} Pc=\overset{\infty }{\underset{\tau }{\int }}{\left[\Phi \left(x/\sigma \right)\right]}^{M-1}\;\phi \left[\left(x-s\right)/\sigma \right]dx\\ \;\;\;\;\;\;\;\;+\frac{1}{M}{\left[\Phi \left(\tau /\sigma \right)\right]}^{M-1}\;\Phi \left[\left(\tau -s\right)/\sigma \right], \end{array}$$

where σ = (σ_int_^2^ + σ_ext_^2^)^1/2^.

**Figure 1:**
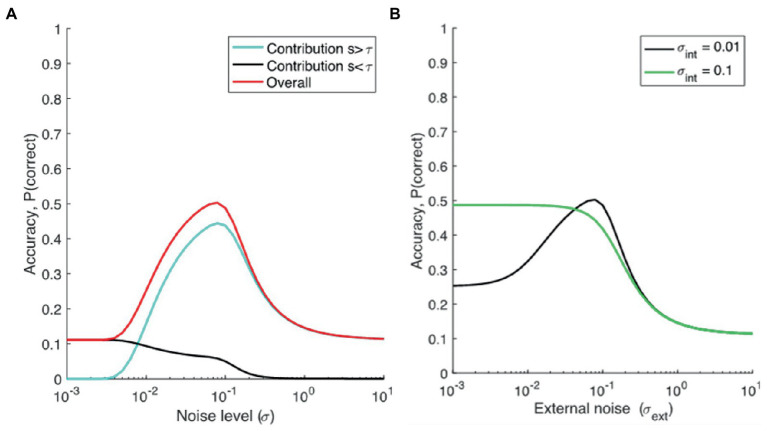
**(A)** Modeling proportion correct dependent on noise. *M* = 9, τ = 0.2, *s* = 0.19, σ_int_ = 0, σ_ext_ is varied, and **(B)** the dependence of accuracy (*Pc*) on external noise (σ_ext_), assuming different levels of internal noise for two groups, a low-noise group (representing TD individuals, or TD individuals with lower AQ scores, σ_int_ = 0.01), and a high noise group (representing ASD, or TD individuals with higher AQ scores, σ_int_ = 0.1).

Figure 1A shows the contribution of the two parts of the equation (i.e., target signal above threshold, and guesses), dependent on external noise alone (setting internal noise to zero), when *M* = 9. When noise is low, the signal plus noise will be below threshold (because *s* < τ), and so will the *M*-1 non-target alternatives (as their mean is centered at 0). Therefore, the proportion correct depends completely on guesses, and equals 1/*M*. When noise is higher, the signal plus noise will pass the threshold more regularly than the alternatives (because it is offset by the value *s,* and closer to τ), and proportion correct will increase. When noise increases further, noise becomes so large relative to the signal that the signal plus noise is largest only in about 1/*M* of the trials. Proportion correct then will revert back to chance level.

We can now compare cases with high and low internal noise. As seen from Figure 1B, we can see that the model predicts superior performance at low external noise levels for individuals with larger internal noise, possibly explaining superior performance reported in ASDs on some tasks, and possibly in higher vs. lower AQ groups (in TD individuals). Performance for the same group declines when noise is further increased. This decline in performance at higher noise levels is also more exaggerated for the higher AQ scores group (or in ASD groups) than TD individuals with lower AQ scores. Also, we can see that the model predicts SR for lower AQ (or TD) participants but smaller or absent SR for higher AQ (or ASD) participants.^2^ The results from this model aligns with the SR hypothesis of Simmons et al. (2009), and the high neural noise hypothesis for ASD in general. Note that, for the experiments reported here, we have comparisons between lower and higher AQ groups, but the model makes the same predictions for TD and ASD groups.

## 3. Experiment 1

In the first experiment, we conducted a lab-based visual detection task based on the approach of Itzcovich et al. (2017). We used alphabetical letters as stimuli in the task, because of the potential relevance of this task in daily living. We also collected AQ measures for each participant, to determine whether SR parameters (such as the position of the peak) depend on autistic traits.

### 3.1. Materials and methods

#### 3.1.1. Participants

A total of 33 participants participated (10 males, 23 females, *M_age_ =* 25.4*, SD_age_ = *9.50). Of these, 26 participants identified as University of Canberra students and seven did not. All had normal or corrected to normal vision. The research received approval from the University of Canberra Human Research Ethics Committee (ethics number: 1868) and participants received a written information sheet and signed a consent form prior to commencing the experiment. All participants were reminded that they could withdraw at any time and were given contact information of the experimenter so they could ask any questions or raise any concerns they may have had after leaving the experiment. After data-screening (see below) 24 participants (7 males, 17 females, *M_age_ =* 24.8*, SD_age_ =* 9.18) were included in the analysis. This sample size was based on previous studies that investigated SR using letter identification tasks (Piana et al., 2000; Itzcovich et al., 2017).

#### 3.1.2. Equipment and physical setup

Stimuli were presented on a gamma-corrected Dell LCD screen (60 Hz, 1920×1080 pixels), which was viewed at an approximate distance of 57 cm without a chin or head rest.

#### 3.1.3. Quest procedure to determine initial letter contrast

The experiment started with two brief sessions in which we used the QUEST (Watson and Pelli, 1983) to establish detection thresholds (75% correct) in conditions without noise. On different trials, one of two letters, D and C, were presented in Sloan font with different white levels on a gray background. The participant had to decide whether a C or D was presented after each trial. Each staircase consisted of 40 trials, and the threshold was taken as the value provided by QuestMean at the end of the staircase. The threshold procedure was repeated twice to determine the level of contrast that resulted in threshold-level performance. The average of the two estimates was taken as the detection threshold.

#### 3.1.4. Visual stimuli

The target stimuli were vertically orientated alphabetical letters C, D, H, K, N, O, R, S, V, and Z, presented in Sloan font. The size of the letters was set at 64 pt. (85.33 px). The stimuli were presented on a uniform gray background and were programmed to be positioned at the center of the screen.

Noise stimuli were generated in MATLAB as separate frames (images). Noise was placed on top of the letter and the gray background, and noise and letter luminance were added. The strength of the noise was varied by varying the standard deviations of the gaussian distribution (i.e., white noise) of the luminance noise. The check sizes of the noise were 1px. The noise images were created anew for each trial and displayed in order at a refresh rate of 60 Hz.

#### 3.1.5. Experimental visual display settings

After participants completed the QUEST procedure the visual experiment began. At this point the experimenter left the room and the participant completed the main visual experiment, and the AQ questionnaire. The stimulus size in the task was around two degrees of visual angle.

The experimental conditions were created in MATLAB and were based on the procedures conducted in Itzcovich et al. (2017). Background luminance was kept at an intermediate gray level (128/255). Each letter was displayed in the center of the screen within a box frame. The luminance of the display letters was higher than the background but differed for each participant based on the QUEST procedure described above. On different trials, one of the 10 letters was displayed within one of seven levels of Gaussian additive noise, with sigma (σ) of 0, 6, 12, 18, 30, 60, and 90. We will refer to this noise as external noise. During the experiment each letter was randomly displayed 14 times, twice for each noise level. The letter was displayed for 1 sec. Participants had to type the letter they perceived on a keyboard, which had the 10 response options highlighted. There was no time limit for the participants to respond. Participants completed 140 trials in the experiment, and the whole experiment took 45 min to complete.

#### 3.1.6. Autism-spectrum quotient

The Autism-Spectrum Quotient (AQ) is a self-administered questionnaire which consists of 50 statements to each of which a respondent must indicate their level of agreement (Baron-Cohen et al., 2001). All items from the original scale were included without any modifications, and were presented on the computer monitor, one by one, in order. Participants selected their answer with the arrow keys, and pressed space to advance to the next question. Participants were required to indicate their responses on a Likert-scale ranging from 1 (definitely agree) to 4 (definitely disagree). The overall scale has a moderate to high internal consistency with Cronbach’s alpha score ranging from 0.63 to 0.77 (Baron-Cohen et al., 2001). This is consistent with findings from other authors (Austin, 2005; Hoekstra et al., 2008; Kloosterman et al., 2011). The AQ was not used as a screening or diagnostic tool for ASD in this manuscript, hence we did not consider clinical score cut offs.

#### 3.1.7. Data screening

Prior to conducting the analyzes, the proportion correct data from each participant were checked for any unusual responses or patterns. We mainly looked for patterns that indicated response bias (such as participants responding with the same answer throughout the experiment), completing the experiment in an unrealistic time, and taking prolonged breaks during the experiment. We found no such unusual responses or patterns (in this experiment or the next). Participants who had 100% correct response at the lowest noise level (zero noise level) were excluded (i.e., nine participants from each study were excluded), because their data would preclude the possibility of showing any increase in performance due to noise. The reason that some participants showed 100% correct at zero noise level can be attributed to the staircase procedure, which may have not worked efficiently, leaving the performance for these participants at a high level. Participants’ data were also excluded if overall performance was not above chance level (1/*M*). For this first experiment, all participants scored above chance level. Lastly, there were no missing values for any of the items.

#### 3.1.8. Data processing and statistics

Proportion correct data were analyzed with the statistics software (The Jamovi Project, 2021) and (JASP Team, 2022). Analysis that required statistical contrasts analysis were conducted in JASP. Note that we have used Generalized Linear Mixed Model (or GLMM) for most of our statistics as (1) our dependent variables are either binary responses or not normally distributed, and (2) the advantage of such models is that it allows us to incorporate random effects factors. As is standard for binomial distributions, we used a GLM with binomial distribution, and logit link functions. We have also provided *OR (odds ratio)* for some analysis. The further the *OR* is from 1, the more likely that there is an association between the variables.

### 3.2. Results

#### 3.2.1. The stochastic resonance effect

**Figure 2:**
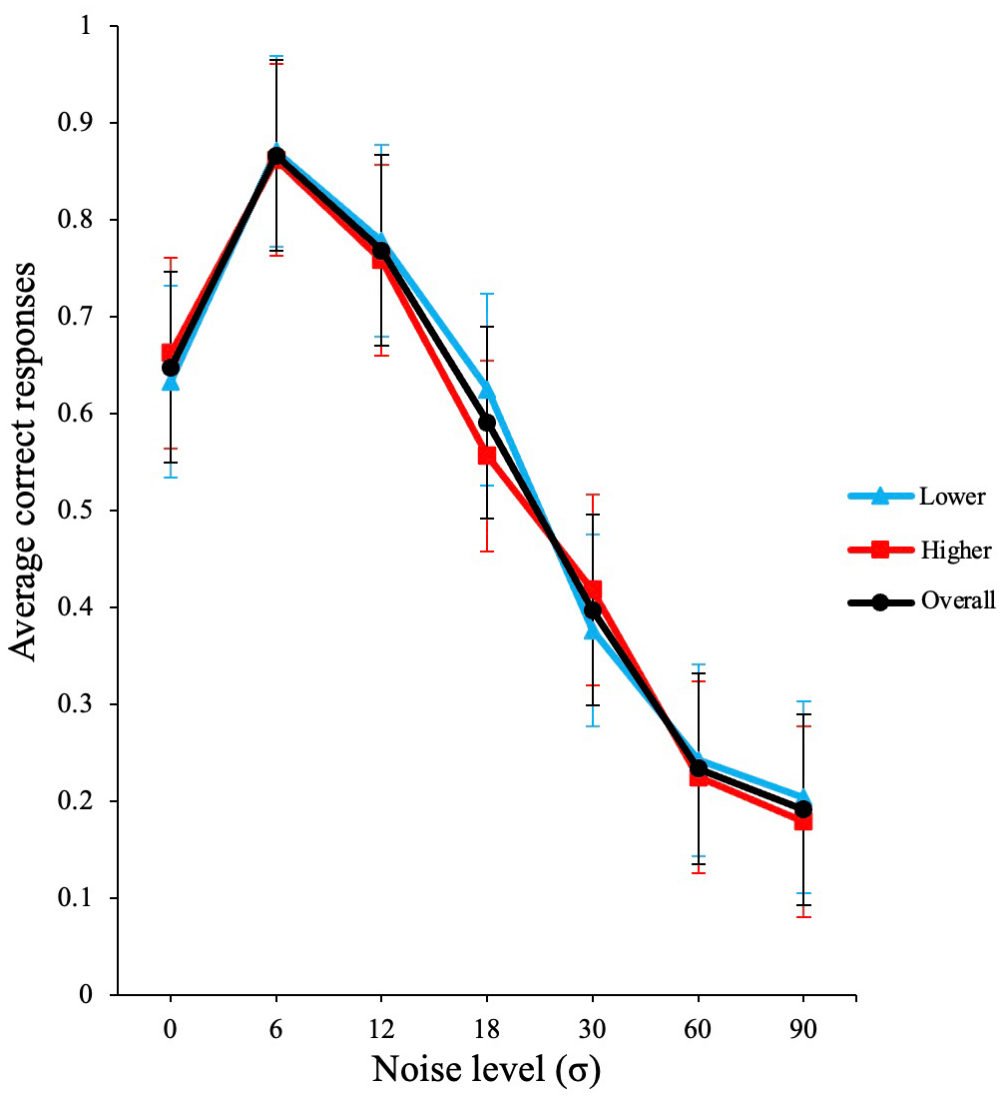
Inverted u-shaped function (SR effect) from Experiment 1. The graph illustrates the raw average proportion correct versus σ noise level. Error bars show standard errors of the means over participants. The blue line illustrates lower AQ participants, and red line shows higher AQ participants.

Our results show clear SR (see black line in Figure 2), with optimal performance occurring at non-zero levels of noise (here *σ* = 6).

A GLMM with binomial family and logit link function was conducted in the JASP software to evaluate the effects of visual noise on the average correct responses of participants. The participants were used as a random effect grouping factor and noise was used as the fixed factor. The analysis produced a significant main effect for external noise level σ [*χ*^2^ (1) = 722.88, *p* < 0.001, *VS*-MPR (Vovk-Sellke Maximum *p*-ratio) = 3.18 × 10^155^].

Next, we conducted a planned contrast analysis. A planned contrast analysis revealed that the increase in performance from zero noise was significant at *σ* = 6, *z*(∞) = 7.01, *p* < 0.001. When further external noise is added (*σ* =12), performance starts to decline as observed in Figure 2. This decline in performance was also significant in the contrast analysis; *z*(∞) = −3.81, *p* < 0.001. The planned contrast p values were Holm-Bonferroni adjusted.

#### 3.2.2. Dependence on AQ traits

To determine the impact of AQ, we categorized AQ scores into higher and lower AQ scores. There is evidence that indicates that the most accepted form of categorization of autistic traits is by performing a median-split (Stevenson and Hart, 2017). Therefore, the median-split approach was used for categorizing our group. The median in this data was 20.50, and hence participants that scored below 20.50 were considered lower AQ (*N =* 12, *M =* 15.08, SD = 3.75, Range: 7–20) and participants that scored 20.50 and above were deemed as higher AQ (*N =* 12, *M =* 24.50, SD = 3.52, Range: 21–34), see blue and red lines in Figure 2. Note that, we do not actually have many AQ scores above 32 (a cut-off when AQ is used for screening), and the group means are well below 32. Therefore, we chose to name the groups lower and higher AQ groups (and not low and high AQ groups). A GLMM with binomial family and logit link function showed a non-significant main effect for AQ group [*χ*^2^ (1) *=* 0.05*, p = *0.82, *OR* = 1.07], a significant effect for noise level (σ) [*χ*^2^ (1) *=* 263.51*, p < *0.001, *OR* = 0.97], and a non-significant interaction for noise level (σ) and AQ group [*χ*^2^ (1) *=* 1.43*, p = *0.23, *OR* = 1.00]. This analysis shows that there was no significant difference in performance between the higher and lower AQ groups, who showed similar levels of SR.

#### 3.2.3. Improved detection threshold in participants was not linked to autistic traits

As a check whether there were any differences in detection thresholds (as measured with QUEST) between individuals with lower and higher levels of AQ, we performed a linear mixed model. We checked for outliers using Z-scores on the threshold values, revealing one outlier (Z-score *>* 3.29). Furthermore, when we fitted the linear mixed model with all participants included, and checked the residual and predicted value plots, the participant’s residual was an outlier as well. Therefore, this participant was excluded from the main analysis.

A GLMM with Gamma distribution, and inverse link function was conducted with the threshold values as the dependent variable, AQ score was included as a covariate, and the order of the two threshold sessions was a fixed factor. The participants were added as the random effects in the model.

The results from the analysis yielded a significant effect of order [*χ*^2^ (1) *=* 4.83*, p = *0.03, *OR* = 1.12], a non-significant effect of AQ [*χ*^2^ (1) *=* 1.41*, p = *0.24, *OR* = 1.00], and a non-significant interaction effect of order and AQ [*χ*^2^ (1) *=* 0.28*, p = *0.60, *OR* = 1.00]. This analysis indicates that participants on average performed better in the second threshold procedure, but this (improvement in) threshold score was not linked to their AQ scores (Supplementary Figure S1).

#### 3.2.4. Impact of letters

**Figure 3:**
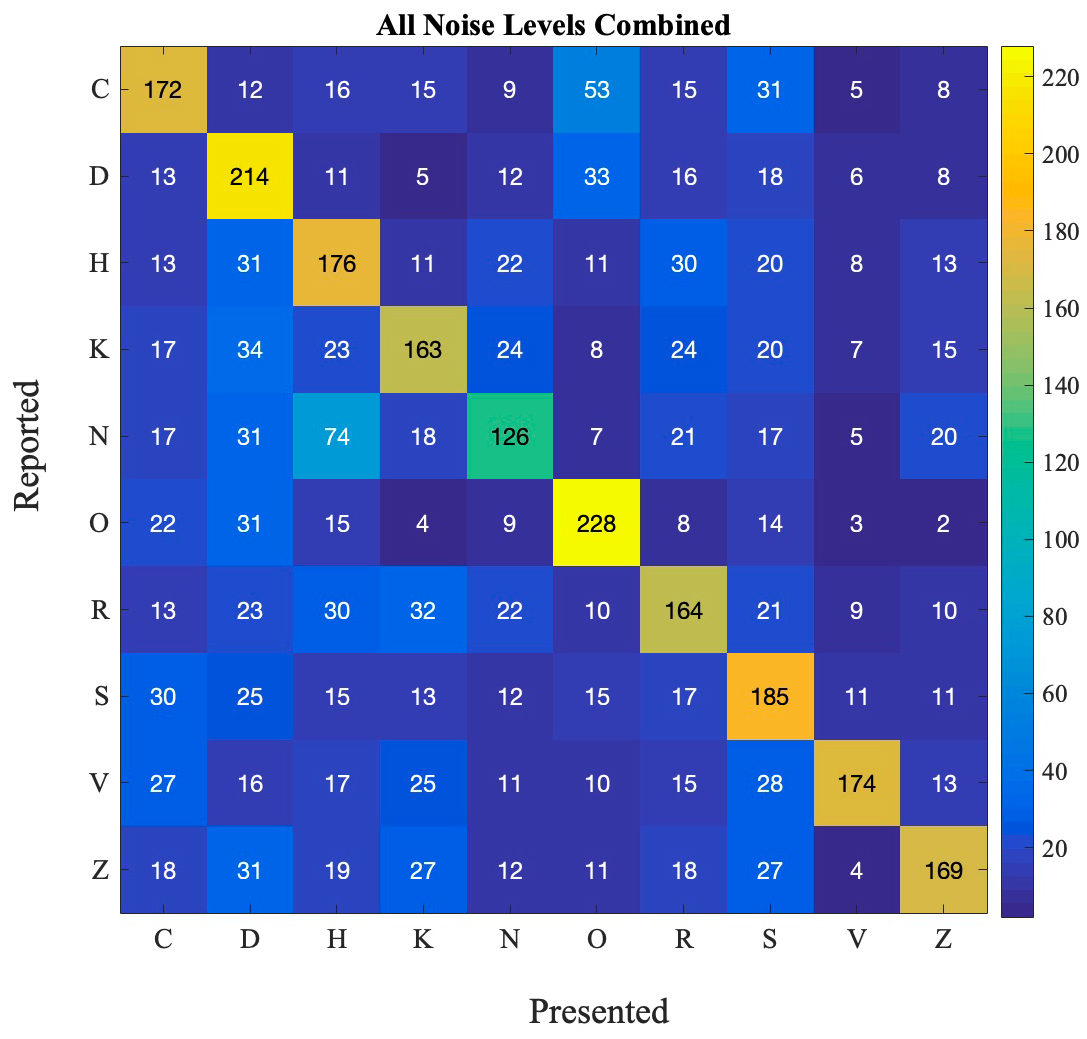
Confusion matrix depicting the frequency of reported and presented letters in Experiment 1.

SR effects may be different between different letters, because some letters are more confusable than others. We computed a confusion matrix (Figure 3) showing for each presented letter (*x*-axis), which letter was reported (*y*-axis), to see how participants performed for each letter in the detection task. This analysis allows us to investigate how different stimuli can impact the occurance of SR. This can also have real-life implications as there are numerous stimuli present in the environment. Thus, for the SR-phenomenon to be effective in ecological settings, it has to work with different stimuli. Furthermore, if some letters are very easy to identify, and accuracy is close to 100% for that letter, SR would probably be too small to measure.

From the matrix, we can see that some letters are often confused, such as R and K, O, C and D, and H and N. Other letters are not as often confused with other letters (e.g., O and K). This happens at all noise levels (also see Supplementary Figure S2 for performance at each noise level).

**Figure 4:**
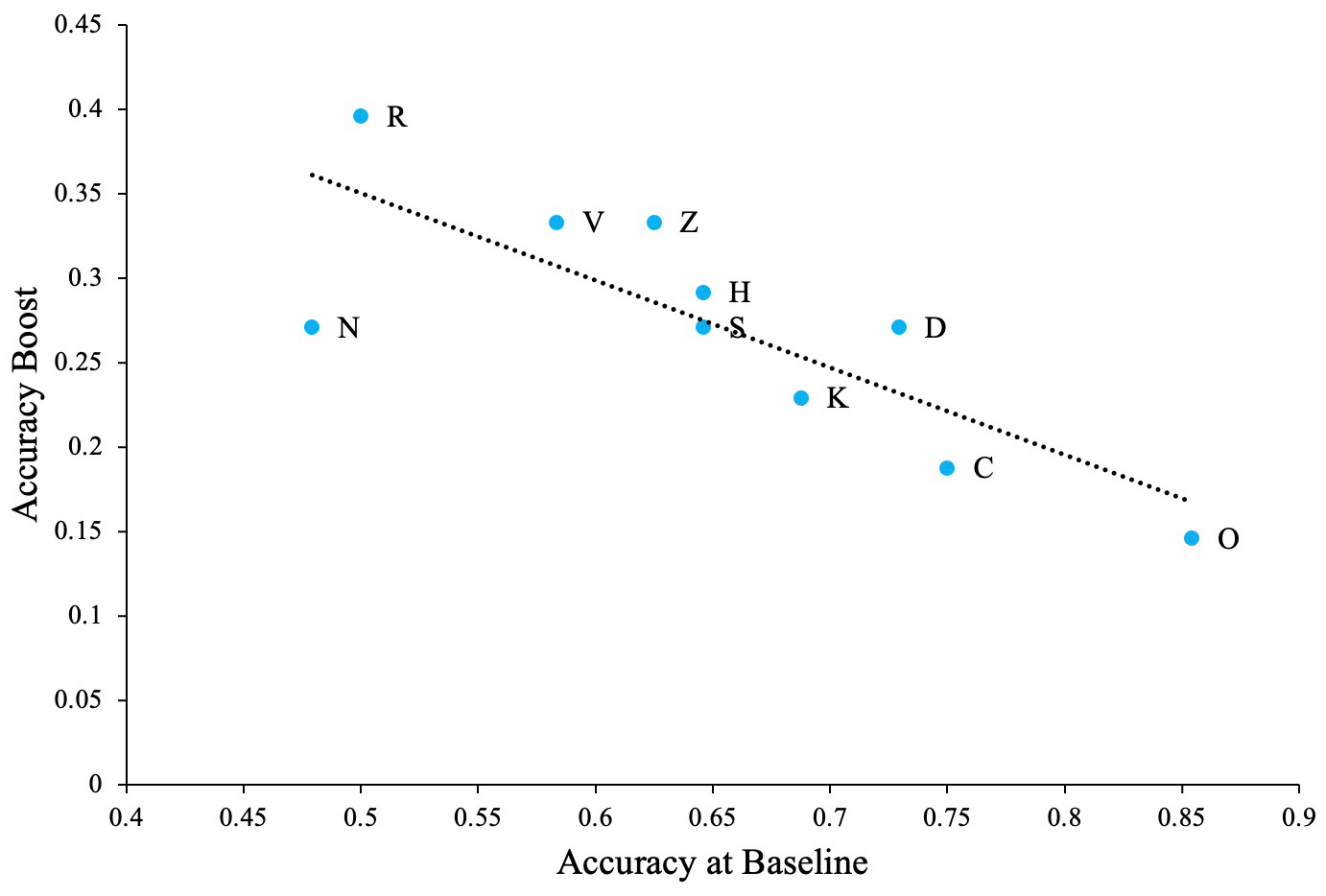
The impact of SR when the task contains confusable letters. Dashed line Indicates the regression line (trendline). Plot shows negative association between baseline accuracy and improvement in accuracy above baseline (accuracy boost). The baseline is accuracy of participants at zero external noise level.

We believe that when such confusable letters are involved in the task, detection rates will be lower at zero noise levels allowing the effect of external noise and SR to be more prominent. To test this hypothesis, we first calculated the difference between zero noise and the maximum performance at any non-zero noise level (we refer to this as ‘accuracy boost’) for all participants. Overall average performance at zero noise for all letters (and all participants) is negatively correlated with the accuray boost as seen in Figure 4 (Pearson’s *r* = −0.80, *p* = 0.005). This plot indicates that when accuracy at zero noise is low, the beneficial impact of noise is high and vice versa.

**Figure 5:**
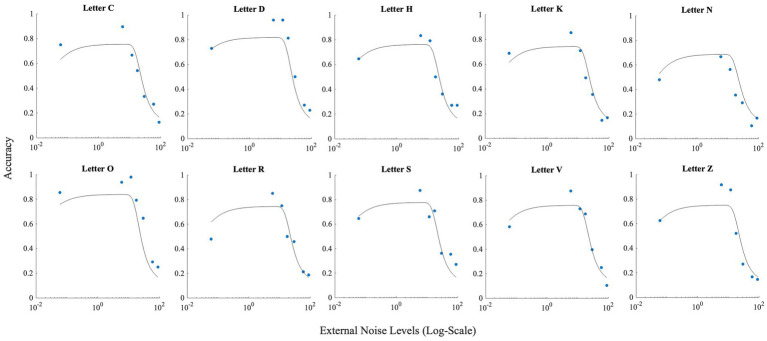
This figure illustrates the effect of noise on each letter. Horizontal axis (*x*-axis) represents noise levels in log-scale, and the vertical axis (*y*-axis) shows accuracy. Accuracy at zero external noise was plotted at a level of 6 × 10^−2^ of external noise. Lines show the fit of the model (Model 1) to this data.

These differences between letters notwithstanding, all letters showed the inverted U-shaped function indicating strong presence of SR (Figure 5; data points).

## 4. Experiment 2 (online experiment)

A shortcoming of the first experiment is that we could not estimate internal noise measures for the participants (due to the low number of trials) and correlate it with AQ scores. Here, we performed another visual identification experiment, which had several strengths. First, we ensured that we had more (12) repetitions per combination of noise level and letter identity, which allowed us to estimate internal noise measures for each participant and correlate these measures with AQ scores. Second, due to the COVID-19 pandemic, we had to conduct this experiment online. This forced us to look at whether SR can occur in a more ecologically valid scenario of online letter identification, instead of using a lab-based setting. Third, we expanded the ecological relevance by using the first 9 letters of the alphabet, instead of a carefully curated set of letters as typically done. Fourth, we did not only include white noise, but also three types of colored noise. We used colored noise as most behavioral studies investigating SR have only used white noise to show SR. We wanted to investigate if colored noise could produce a better SR effect than traditional white noise as suggested by some previous research in another context (Duan et al., 2014).

### 4.1. Methods and procedure

#### 4.1.1. Participants

Participants in this study were 48 undergraduate students (24 males, 24 females, *M_age_ =* 21.10*, SD_age_ =* 4.63) from the University of Canberra, who had normal or corrected to normal vision, and were undertaking an introductory psychology unit. All participants were aged 18 and above. This research was approved by the University of Canberra Human Research Committee (ethics number: 4454). After data screening (see below), 30 participants were included in the analysis (13 males, 17 females, *M_age_ =* 20.37*, SD_age_ =* 3.49). This sample size was again satisfactory based on previous studies that investigated SR using letter identification tasks (Piana et al., 2000; Itzcovich et al., 2017).

#### 4.1.2. Stimulus and measures

The visual stimuli were presented using Gorilla Experiment Builder.^3^ Gorilla is a cloud software program designed specifically for behavioral sciences. Target stimuli were vertically orientated alphabetic letters ranging from A to I, presented in Arial font. The size of the letters was set at 300px. The stimuli were presented on a uniform gray background and were programmed to be positioned at the center of the screen.

Noise stimuli were generated in MATLAB as separate frames (images). These noise images were placed on top of the letter and the gray background. The strength of the noise was varied by varying the opacity of the noise stimuli. The size of the noise stimuli were 344 × 344 pixels, and 72 pixels/inch. There were four different types of noise, white noise (flat spatial and temporal frequency spectrum; 1/*f^n^* with *n* = 0), and noises with decreasing amounts of high spatial and temporal frequencies: pink (*n* = 1), brown (*n* = 2), and a noise we will call infrared noise (*n* = 4). All types of noise were created as one series of images, saved in a PNG format. They were then loaded into the Gorilla program and displayed in order at a refresh rate of 60 Hz. There was one sequence of noise for each noise color that was reused throughout the experiment.

To provide the best performance, participants were restricted to perform this experiment on a desktop or laptop computer. Participants were advised to perform this experiment with minimum distractions (for example, switching off their phone notifications).

#### 4.1.3. Procedure

Participants had to consent to participate in the study, and consent for their data to be used in future studies. Participants then provided basic demographic information, such as gender and age. They were then provided with instructions explaining what they were required to do to successfully complete the experiment.

**Figure 6:**
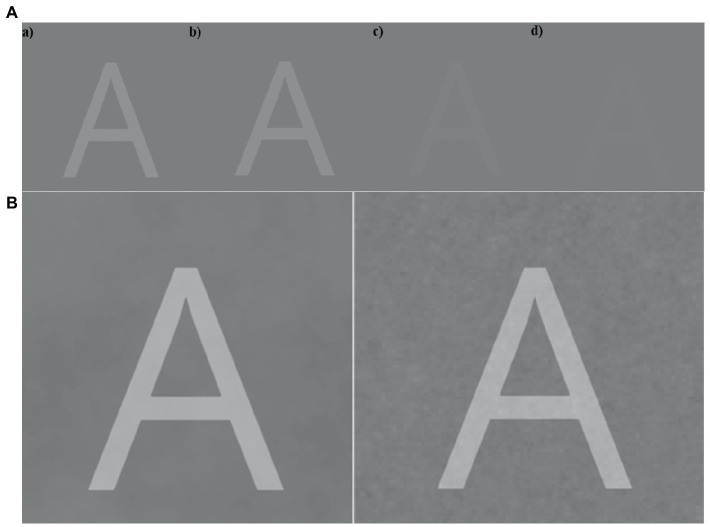
**(A)** Example of the progression in the staircase procedure with the stimulus letter ‘A’. Image (d) illustrates an example when the letter A is about at the visual threshold. **(B)** Example of a stimulus presented with different types of background noise (Brown on the left versus Pink on the right) for the main task. In the experimental setting, the letters were displayed with lower intensities.

Before initiating the main experiment, participants completed a staircase procedure, which served as a practice procedure for the participants, but was also a crucial aspect for our main experiment. For the staircase experiment, participants were shown a series of letters which started with the contrast of the letters being high (letter was light gray), allowing the participant to observe the letter easily. No noise was presented. After the stimulus was presented and removed from the screen, the participants indicated which letter they perceived. The answer screen consisted of 3 × 3 grid with all possible targets presented (in alphabetical order). The participants selected their answer with a mouse click. A staircase procedure was used to find the visual threshold for the participant (see example in Figure 6A). The threshold was set at 50% correct responses, lowered from 75% correct in the lab-based experiment, to decrease the chances of ceiling performance. The staircase procedure consisted of 45 trials. During the main experiment, stimuli were presented at 95% of the stimulus contrast obtained from the staircase procedure.

In the main experiment, each trial contained one of the target letters (‘A’ to ‘I’), counterbalanced over trials. On each trial, the target letter was shown for 1 sec. Along with these letters, a visual noise was added simultaneously on top of the letter. In this experiment, we changed both the amplitude of noise, as well as the type of noise. The amplitude of noise was changed by changing the opacity of noise field that was displayed. There were five different types of noise levels (see examples in Figure 6B). The opacity levels of the noise were 0.00, 0.003, 0.01, 0.03, and 0.10. Participants gave their answer using the same answer screen as in the staircase procedure. If they were unable to identify the letter, they were encouraged to guess. The participants performed 540 trials (nine letters, four noise colors, five noise levels, and three repeats), taking about 45 min. After the experiment, they were asked to complete an Autism Quotient questionnaire which took roughly 10 min to complete.

#### 4.1.4. Data screening

The proportion correct data from the visual identification trials were imported into the statistics software Jamovi version 2.2.2 and JASP version 0.15.0.0. Similar to the first experiment, analyzes that required specific contrasts were conducted in JASP, and other analyzes were conducted in Jamovi.

A generalized linear model (GLM) analysis was conducted for all individual participants to observe overall dependence on external noise. Participants whose data showed no statistically significant dependence on external noise were excluded from the analysis; one participant was excluded based on this criterion.

Also, we found that eight participants had not scored above chance level and were further excluded. Additionally, nine participants also showed 100% accuracy at zero noise level, and hence, they were also excluded. Lastly, there were no missing values for any of the items.

### 4.2. Results

#### 4.2.1. Impact of noise, and stochastic resonance

A GLMM with binomial family and logit link function was conducted in the JASP software to evaluate the effects of colored noise (infrared, brown, pink, and white) and external noise level (opacity levels of 0.00, 0.003, 0.01, 0.03, and 0.1, modeled as a continuous variable) on the accuracy data. Intercepts were included as random effects.

**Figure 7:**
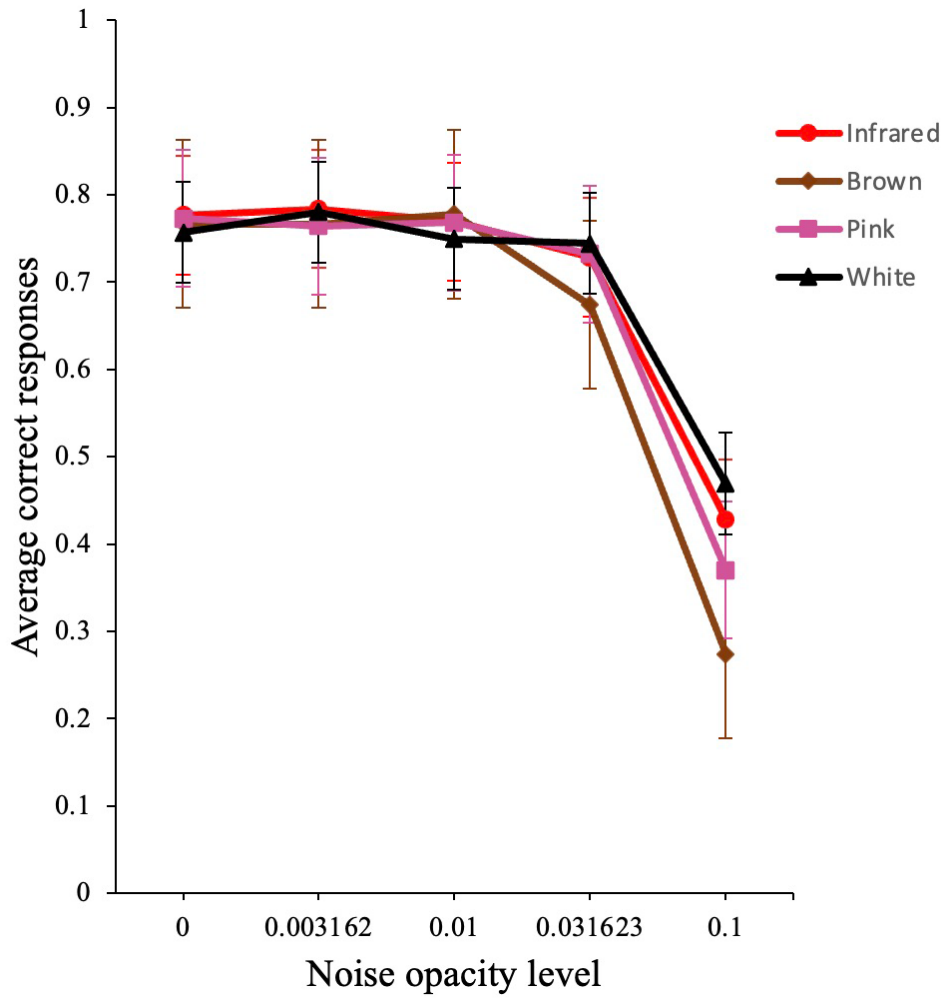
Average performance of participants in the visual detection task. Raw average accuracy versus noise level plot for infrared, brown, pink, and white noise. Error bars show standard errors of the means.

The analysis produced a significant main effect for noise opacity level [*χ*^2^ (1) *=* 1651.76*, p < *0.001, *VS*-MPR = ∞], a non-significant effect for noise color [*χ*^2^ (3) *=* 1.55*, p = *0.67, *VS*-MPR = 1.00], and a significant interaction for noise opacity level and noise color [*χ*^2^ (3) *=* 55.93*, p < *0.001, *VS*-MPR = 3.23 × 10^9^]. The interaction effect reflects the different dependence of external noise for the different colors of noise, especially at higher noise levels (Figure 7).

To investigate the possible presence of SR, we then conducted a planned comparison at two different noise levels for all colored noises: 0.00 versus 0.003, and 0.00 versus 0.01, which we believed to be the optimal levels of noise for this experiment. The analysis revealed that neither comparison was significant (both |*z*| *<* 1.16). Hence, no clear SR effect was found in this version of the experiment.

#### 4.2.2. Superior performance by higher AQ participants

To compare the performance between higher and lower AQ participants, we categorized AQ scores into higher and lower AQ scores. The median in this data set was 20. Participants who scored below 20 were considered lower AQ (*N =* 13, *M =* 14.23, SD = 2.89, Range: 10–19), and participants who scored 20 and above were deemed as higher AQ (*N =* 17, *M =* 23.94, SD = 3.54, Range: 20–31).

A GLMM with binomial family and logit link function was conducted in the Jamovi software to investigate the difference in performance for higher and lower AQ traits group across noise levels in the visual detection task. The participant variable was used as a random effect grouping factor, and the intercept over participants was added as random factor.

**Figure 8:**
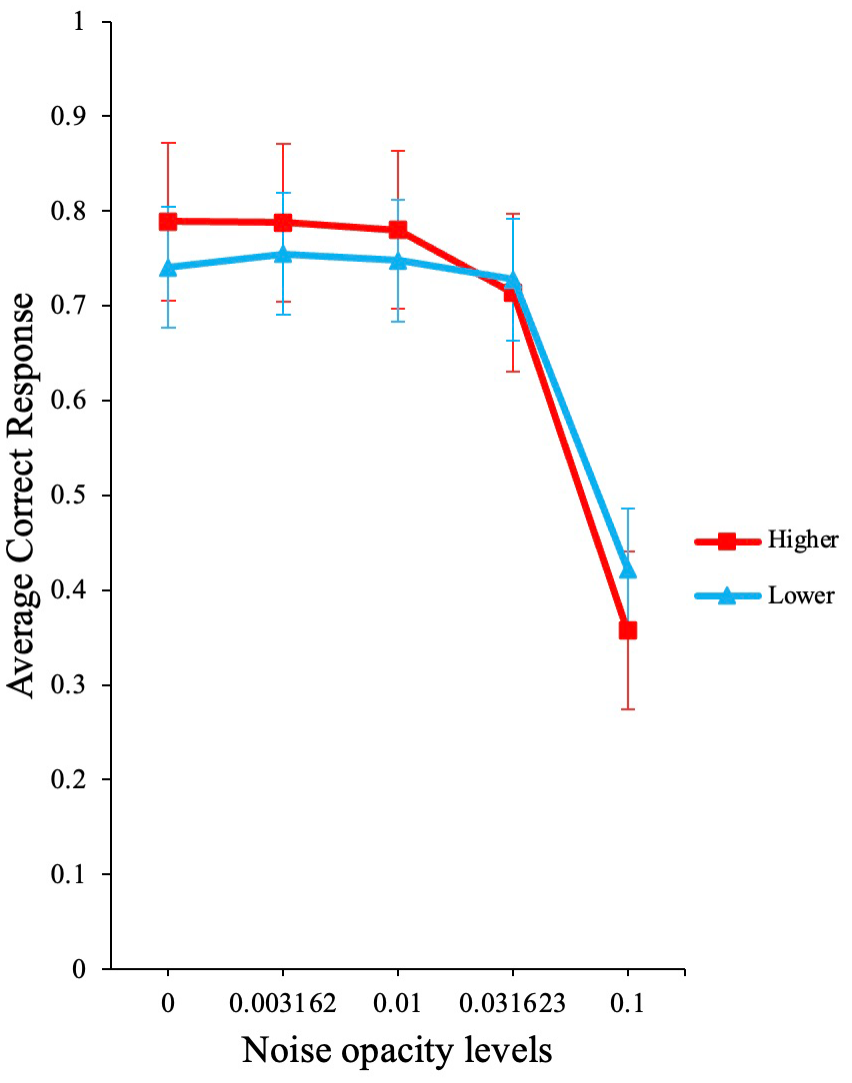
Participant’s performance based on higher and lower AQ. The graph illustrates the raw average accuracies for participants with higher and lower AQ traits. Error bars show standard errors of the means.

The analysis yielded a significant main effect for noise opacity level [*χ*^2^ (1) *=* 1078.15*, p < *0.001, *OR* ≈ 0.00], a non-significant effect for AQ group [*χ*^2^ (1) *=* 0.56*, p = *0.46, *OR* = 0.85], and a significant interaction for noise opacity level and AQ group [*χ*^2^ (1) *=* 20.64*, p < *0.001, *OR* = 76.24]. The average accuracy for the two groups is shown in Figure 8.

These results are consistent with the predictions from our model (Figure 1B). At low external noise levels, participants with higher AQ scores performed better than participants with lower AQ, particularly at zero noise. Also, at higher noise levels, the higher AQ group shows a sharper decline in performance when compared to lower AQ group. These results are also in line with the high neural noise and SR hypothesis for ASD [see (Simmons et al., 2009)].

**Figure 9:**
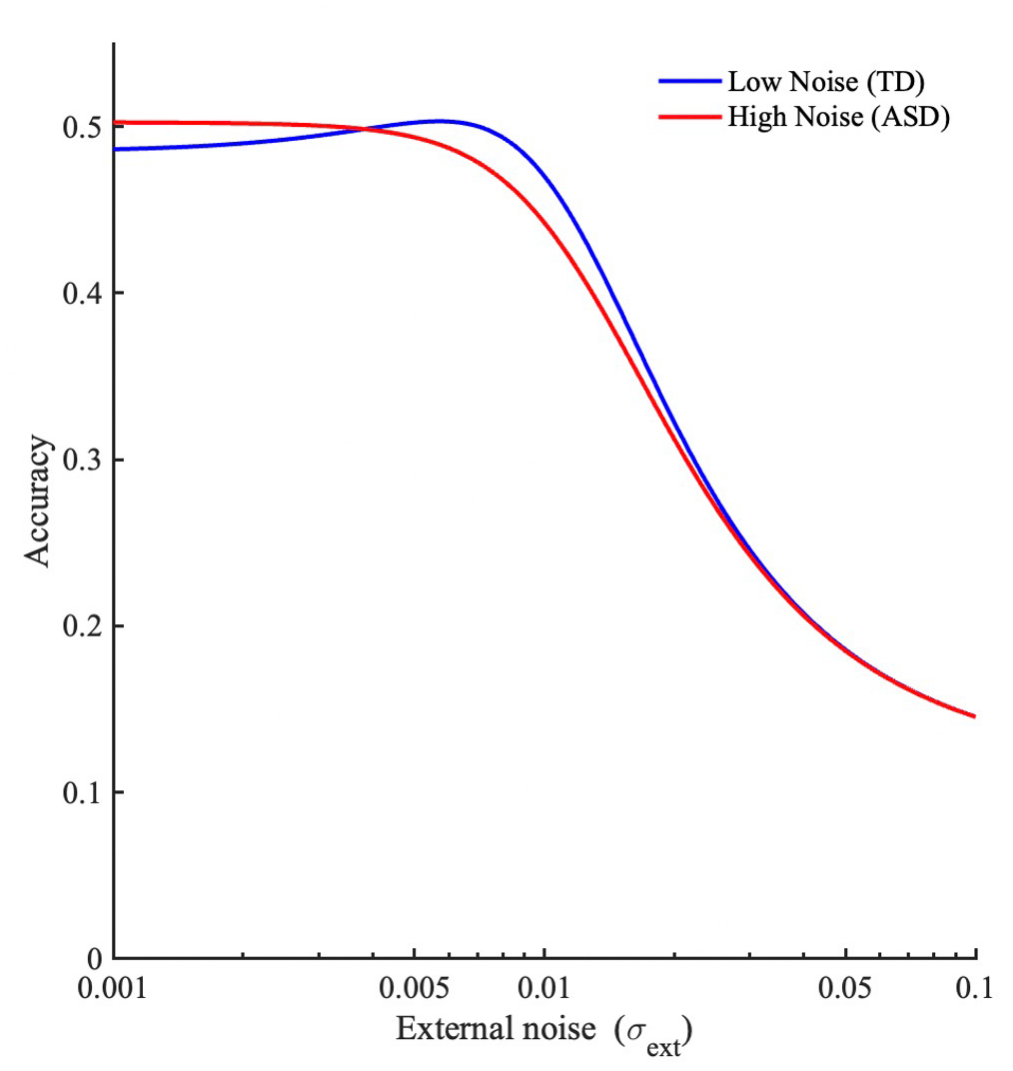
Predictions made by the modeling based on the current experimental parameters.

Our model can more closely capture these effects when we set *M* = 9, *τ* = 0.02, and *s* = 0.95 * τ, to simulate the fact that we set our signal strength in the experiment at 95% of the obtained detection threshold. Then to simulate the difference between the lower and higher AQ group, we set σ_int_ for the lower AQ to 0.005, and for the higher AQ group to 0.008. External noise was varied. With these parameters (Figure 9), the model shows a very similar dependance on external noise to the experimental data. One obvious difference is that the accuracy overall is considerably lower; we will discuss this feature later.

#### 4.2.3. AQ scores did not affect visual detection thresholds

To investigate the relationship between autistic traits and detection thresholds, as measured with the staircase procedure, we employed a generalized linear model with gamma distribution and inverse link function. This revealed a non-significant effect of AQ on detection thresholds [*χ*^2^ (1) *=* 0.11*, p = *0.74, *OR* = 1.00]. Visual inspection on the residuals indicated normality (Q-Q plot, Histogram, and boxplot), and Shapiro–Wilk test also indicated normality (*p = *0.28). The analysis suggests that in the online experiment, autistic traits did not influence detection thresholds in the experiment.

#### 4.2.4. Impact of letters in the online based SR task: Explaining the high accuracy

Accuracy in this experiment was about 75% at zero external noise, which is relatively high for a task with 11% chance level, and also higher than the aimed-for accuracy of 50%. We believe that the high accuracy was at least partly due to the selection of letters (A to I) used. For instance, if we consider the letter A, the outline of the letter A does not overlap with any other letters used from A-I, making it relatively easy to identify, resulting in high accuracy. However, the letter ‘E’ would be more challenging to guess as this letter could be easily confused with the letter ‘F’ and possibly ‘B’, and vice versa.

**Figure 10:**
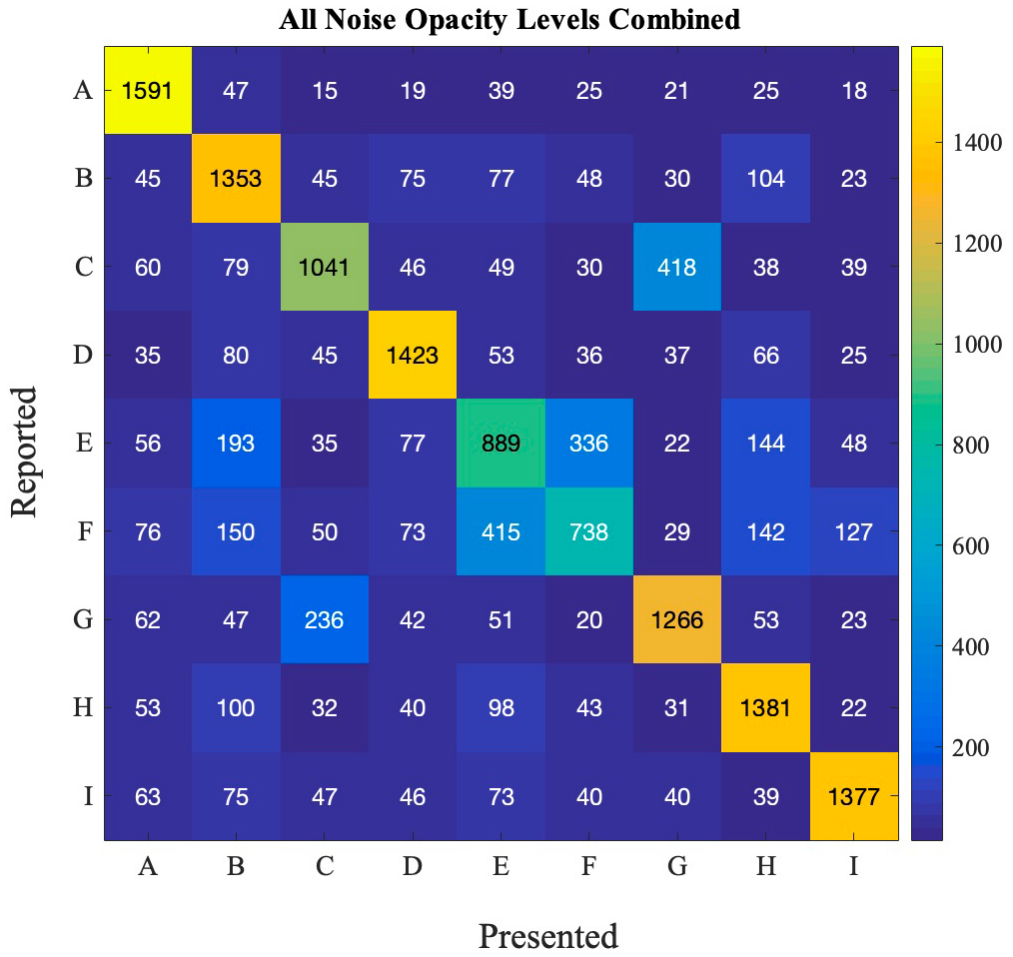
Confusion matrix depicting how accurately the letters in the online experiment were correctly guessed across all noise opacity levels in Experiment 2.

To gage whether there was systematic confusion between letters in this experiment, we computed a confusion matrix. We did this at each external noise level (Supplementary Figure S3) and overall (Figure 10).

Indeed, participants often confused the letter E and F, and also C and G. However, other letters such as ‘A’ or ‘I’ were much easier to guess. This is true across noise levels (Figure 10), as well as noise colors (Supplementary Figure S4). Importantly, even for E and F, they are often confused with each other, but not with other letters, suggesting that guesses were not random, but selected from a smaller subset of letters than the nine possible options.

**Figure 11:**
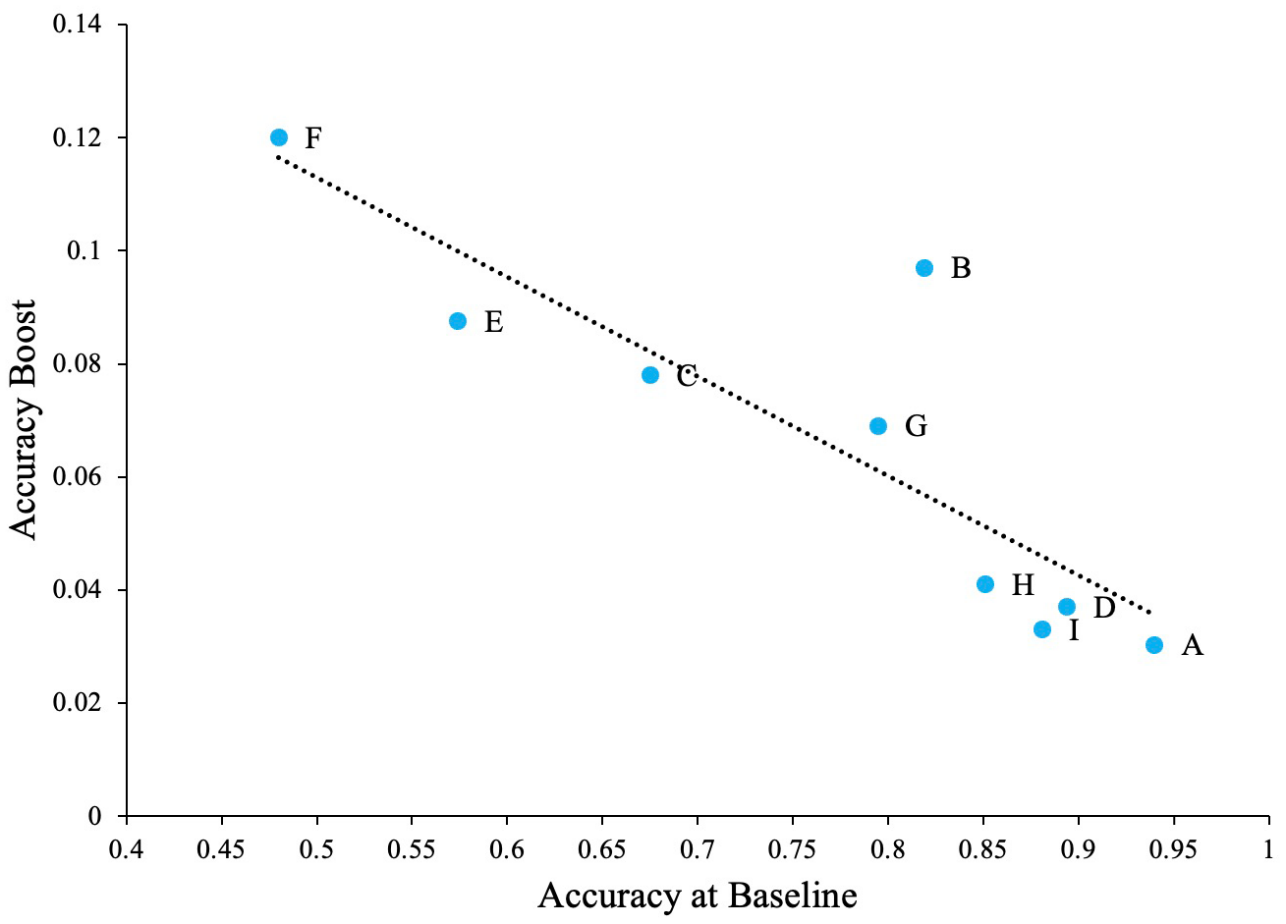
The impact of letters in the SR task when the task contains salient letters (online experiment). Dashed line indicates the regression line (trendline). Plot shows negative association between baseline accuracy and improvement in accuracy above baseline (accuracy boost). The baseline is accuracy of participants at zero external noise level.

**Figure 12:**
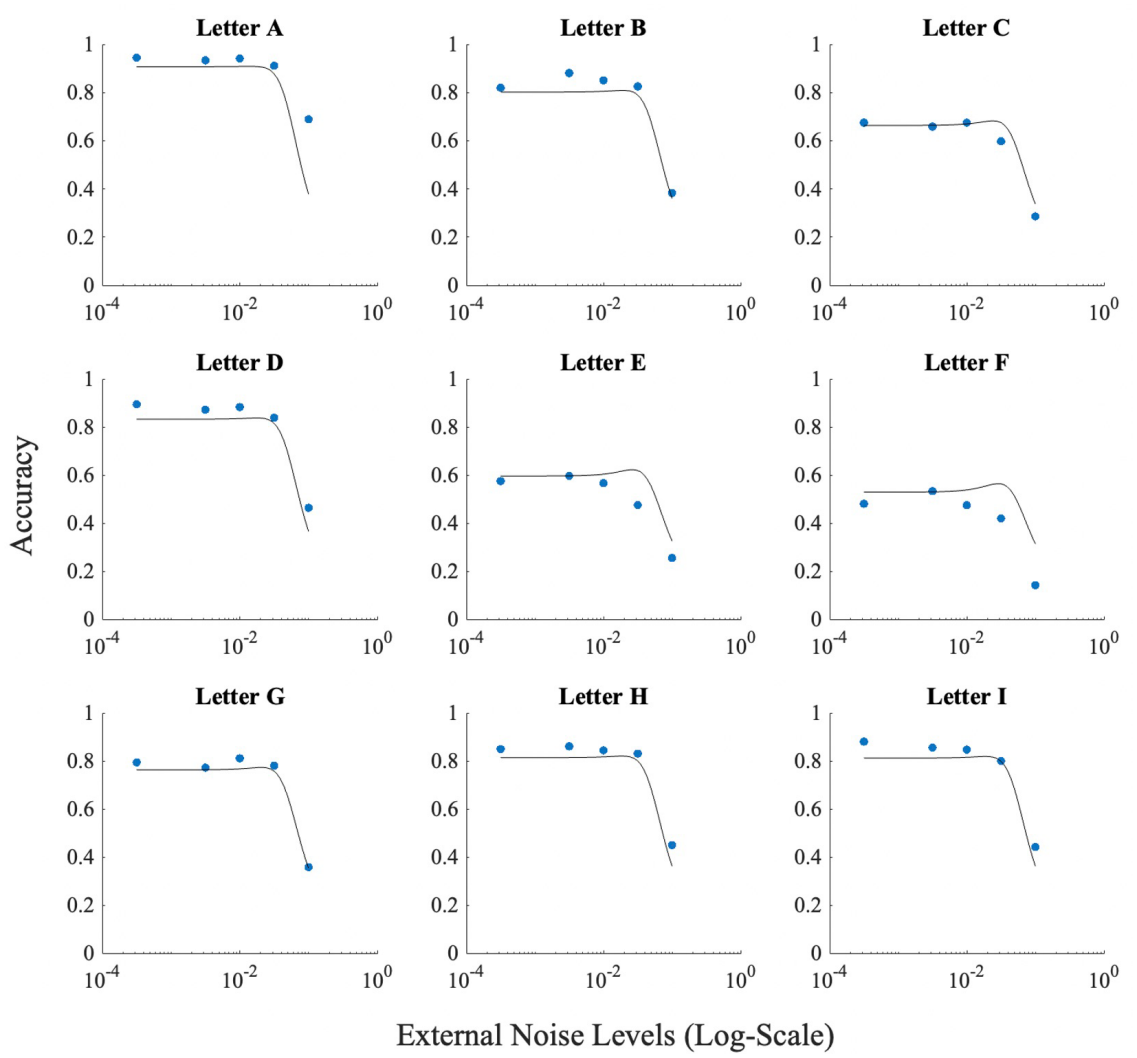
Figure illustrating the effect of noise on each letter used for Experiment 2. Horizontal axis (*x*-axis) represents noise levels in log-scale, and the vertical axis (*y*-axis) shows accuracy. Accuracy at zero external noise was plotted at a level of 3 × 10^−4^ of external noise. Lines show the fit of the model (Model 1) to this data.

The problem with high accuracy levels at zero noise, is that there is little room for improvement by adding noise. Therefore, we looked at whether SR was more pronounced in the letters that were more confusable. Similar to Experiment 1, we plotted accuracy at zero external noise versus the increase of performance relative to zero noise, or accuracy boost, in Figure 11. The correlation analysis showed a ‘very strong’ negative association between baseline accuracy and improvement in accuracy above baseline (Pearson’s *r* = −0.86, *p* = 0.003). From Figure 11, we can see that for more confusable letters such as “E” and “F,” the accuracy was low at zero noise and therefore, the effect of noise, or SR, was more prominent (higher accuracy) when compared to less confusable letters such as “A” or “D” where the zero noise accuracy is high. This trend is also seen in Figure 12, where some SR is seen for confusable letters such as E and F, but not for salient letters like A or D. This figure indicates that SR was present in our data but only for specific letters.

**Figure 13:**
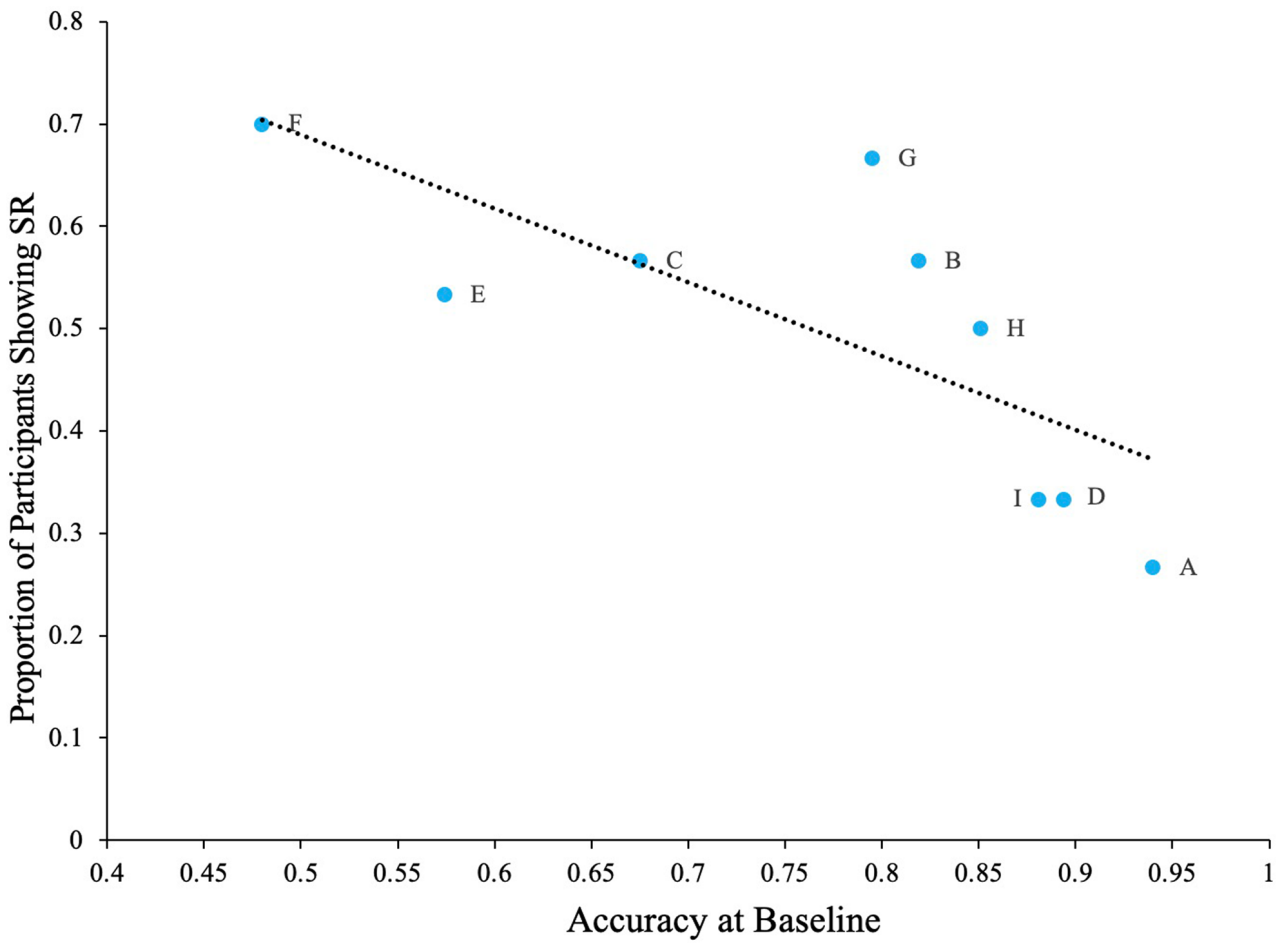
The proportion of participants showing SR for each letter in relation to the letter’s baseline accuracy. Dashed line indicates the regression line (trendline). Plot shows negative association between baseline accuracy and proportion of participants showing SR. The baseline is accuracy of participants at zero external noise level.

Lastly, there was also a negative correlation between the proportion of participants that showed SR for each letter and baseline accuracy for the letter (Pearson’s *r* = −0.74, *p* < 0.05). From Figure 13, we can see that greatest number of participants showed SR for the letter F, which also had the lowest baseline accuracy, and least number of participants showed SR for the letter A, which had the highest baseline accuracy.

Additionally, it is possible that the single staircase procedure in this experiment did not work well for some participants in this version of the experiment. In such instances, the staircase procedure would have ended prematurely at a high contrast level, consequently leading to a high contrast for those participants in the zero noise and other conditions. However, we investigated all staircases, and identified staircases that may not have converged, and those that did converge. The staircases that converged showed a numerically higher accuracy than those that may not have converged, suggesting that the non-convergence of staircases cannot explain the elevated accuracy rates.

## 5. Adjusting the model to allow for higher accuracies at low noise levels

In this section, we will present the modified model which allows us to fit our data where higher accuracies are present at lower noise levels. We used the adjusted model on the overall data, and we also used it on individual data. For all the fits we included lower and upper bounds, and we used the least squares method as the error minimization approach. The lower and upper bound was set to 0 and 100, respectively, for Experiment 1. For Experiment 2, the lower bound was 0 and upper bound was 0.l. All the fitting was done using MATLAB.

To address the high accuracy in the data, we introduced a parameter *M_2_* in the model. *M_2_* like *M* represents the alternatives in the experiment, but it is smaller than *M*, because it represents the number of alternatives that the individual is guessing between when they are not certain, e.g., in the case of letters ‘E’ and ‘F’ which are confused with each other as they have shared or overlapping features. Hence, the value of *M_2_* here will be closer to 2 (as there are two alternatives to choose from). *M_2_* was implemented in the model from Equation (3) as


(6) $$Pc_{s<\tau }=\frac{1}{M_{2}}{\left[\Phi \left(\tau \right)\right]}^{M-1}\;\Phi \left(\tau -s\right).$$

With the introduction of *M_2_* in Equation (6), the final equation of the model, Equation (5), is changed to


(7) $$\begin{array}{l} Pc=\overset{\infty }{\underset{\tau }{\int }}{\left[\Phi \left(x/\sigma \right)\right]}^{M-1}\;\phi \left[\left(x-s\right)/\sigma \right]dx\\ \;\;\;\;\;\;\;\;+\frac{1}{M_{2}}{\left[\Phi \left(\tau /\sigma \right)\right]}^{M-1}\;\Phi \left[\left(\tau -s\right)/\sigma \right]. \end{array}$$

We first fitted the model on the overall data for Experiment 1. The signal strength (*s*), threshold (τ) or criterion, and internal noise were fitted simultaneously. *M* was set to ten, and *M_2_* was obtained from the confusion matrix. To obtain *M_2_*, we used the formula 1/(total number of responses for the presented letter/total number of responses). This produced different *M_2_* values for all letters. The initial value of *s* was set as 2.027 which was approximately the stimulus strength used throughout the experiment. The initial τ, was set at *s*/2, the optimal setting in most signal detection frameworks; this setting does not produce SR. Initial internal noise ( σ _int_) was set at 0.00. Figure 5 depicts the results produced by the fit for Experiment 1. These fits show that adjusting *M_2_* based on the obtained data (from the confusion matrices), leads to approximately correct levels of accuracies higher than 50%.

To check for the presence of SR, we investigated whether the peak of the fitted curve is higher at any non-zeros level of noise, than at the zero-noise level. If the difference was positive, SR was present for the letter. The fit produced SR for all letters with letter ‘O’ having the smallest SR (accuracy boost = 0.08), and letter ‘N’ having the largest SR (accuracy boost = 0.16).

We performed the same fit on the data from Experiment 2. All values for the initial parameters remained the same except the value of *M* was set to nine, and τ was changed to 1.025/28, which was the approximate average stimulus strength used in Experiment 2. Figure 12 illustrates the results produced by the fit for the online experiment. Again, accuracy levels were estimated relatively well, and the fit produced SR for all letters with letter ‘A’ having the smallest SR (accuracy boost = 0.0015), and letter ‘F’ having the largest SR (accuracy boost = 0.04).

### 5.1. Fitting the model to individual participant data

In an effort to estimate internal noise levels from the model, we fitted the model to individual data in Experiment 2, and then correlated the noise level estimated from the fits to AQ scores. Due to low trial numbers in Experiment 1, we were unable to fit the model on individual participants for that experiment.

To obtain the best model for the individual data, we compared multiple models (with different free and fixed parameters) to assess the best fit. The parameters (fixed and fitted) for the first model were the same as the ones described in the previous section (i.e., Model 1 in Supplementary Table S1). In the second model (Model 2 in Supplementary Table S1), the fixed parameter was *M_2_,* and the fitted parameters were τ (fitted independently for all letters), *s* and internal noise (combined over all letters). In the third model (Model 3 in Supplementary Table S1), *M_2_* was fitted separately for each letter, as well as fitting σ, *s*, and internal noise. In the last model (Model 4 in Supplementary Table S1), *s* was kept as the fixed parameter (combined over all letters), and *M_2_* (separately for each letter), τ (independently for all letters), and internal noise (combined over all letters) were the fitted parameters. We used the fit that produced the lowest Akaike information criterion (AIC) value, averaged over all the participants, to present our data here. We have summarized all the models in Supplementary Table S1. Note that the more negative the AIC value, the better the fit.

The AIC values indicated that the best model for fitting individual data was Model 3 (Supplementary Table S1). We used the least squares method for the error minimization approach, and we set the lower bound to [1, 0] and upper bound to [9, 0.1] for this model.

**Figure 14:**
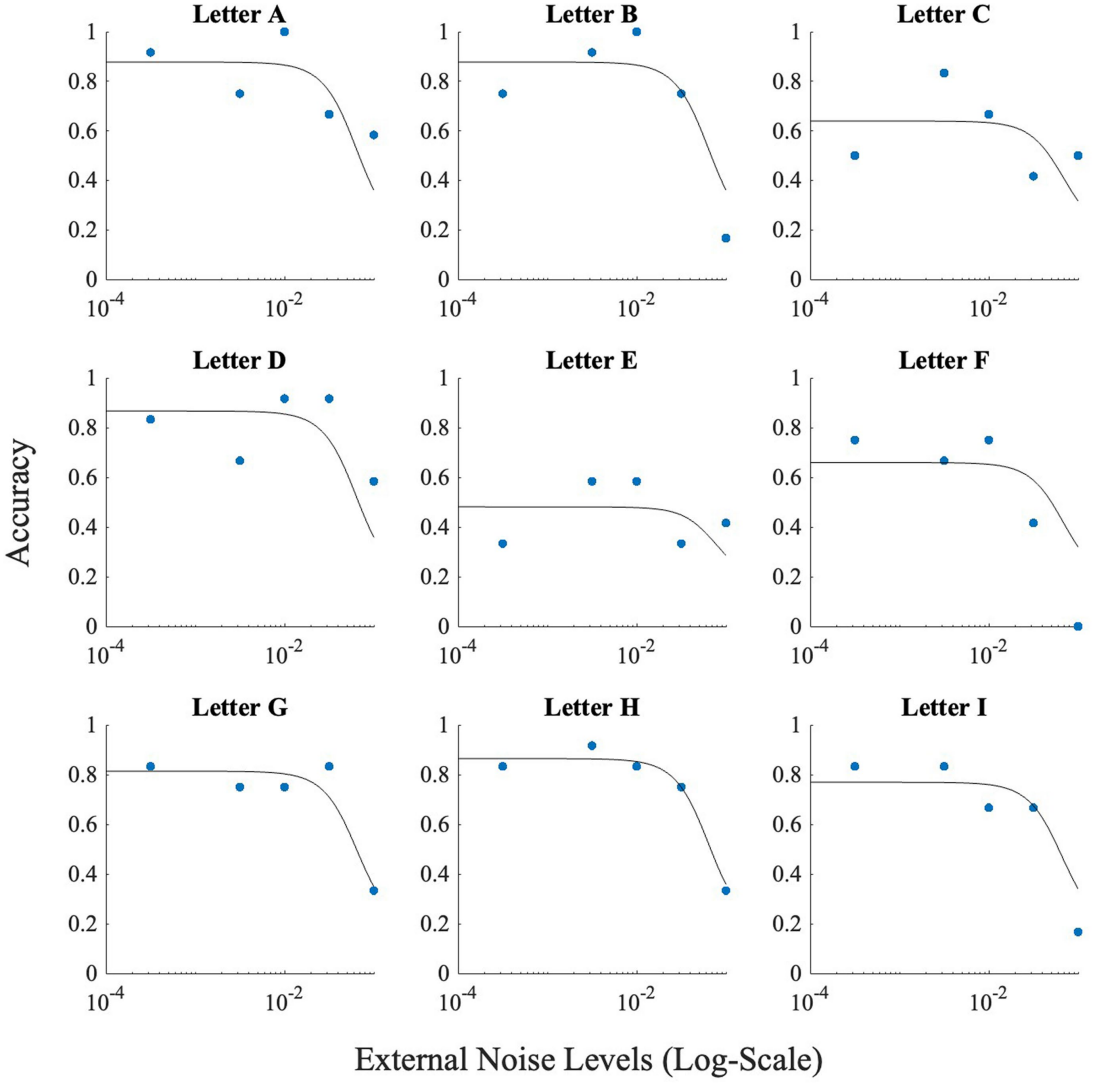
An example fit of Model 3 to one participant. The fit produced no SR for this participant. Horizontal axis (*x*-axis) represents noise levels in log-scale, and the vertical axis (*y*-axis) shows accuracy. Accuracy at zero external noise was plotted at a level of 3 × 10^−4^ of external noise. Lines show the fit of the model (Model 3) to this data.

We also checked SR for all participants (i.e., checked whether the peak of the fitted curve was higher at any non-zeros level of noise), and we found that 12 out of the 30 participants had shown SR. Figure 14 shows an example of the fit for one participant.

**Figure 15:**
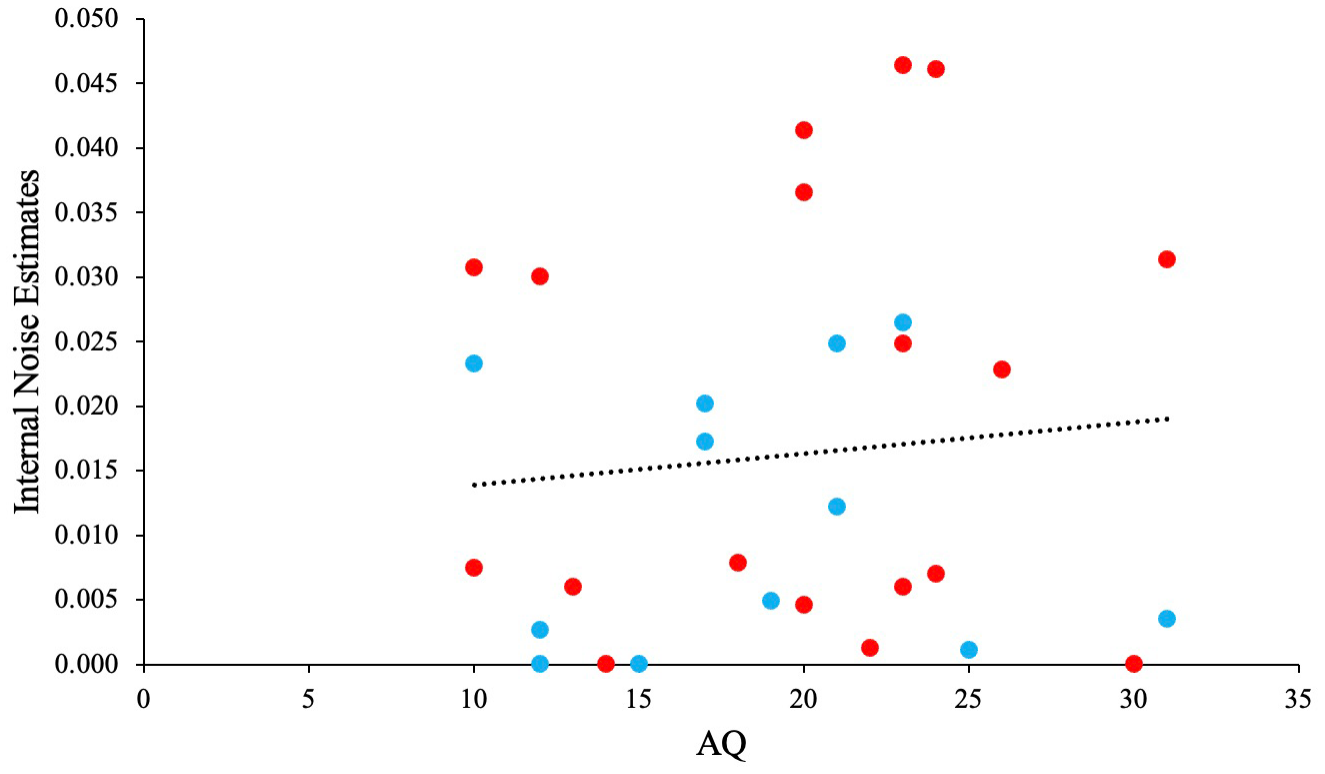
The relationship between AQ and Internal Noise. Blue scatter illustrates participants that showed SR, and red scatter illustrates participants that did not show any SR.

We performed a GLM analysis using the Gaussian distribution and identity link function to investigate the relationship between AQ, and internal noise estimated from the fitted model. Before the analysis we checked for any outliers. No outliers were identified in the data (all *Z* < 3.30). Kurtosis, skewness, Q-Q plot indicated normality for the dependent variable ‘internal noise’. The GLM analysis yielded a non-significant main effect for AQ [*χ*^2^ (1) *=* 0.26*, p = *0.61, *OR* = 1.00], with a slight positive slope. Figure 15 depicts this non-significant trend. When taking the noise estimates from the best model for each participant individually (instead of from the best overall model), the results did not change considerably.

## 6. Discussion

### 6.1. Stochastic resonance in the visual domain

We performed lab-based and online visual-identification-in-noise tasks. We found that SR was clearly present in the laboratory-based task, but not in the online experiment. Our findings from the laboratory experiment are consistent with past findings, showing SR in a variety of visual tasks (Kundu and Sarkar, 2015; Treviño et al., 2016; Van der Groen et al., 2019), and specifically the letter identification used by Itzcovich et al. (2017). Treviño et al. (2016) showed that visual noise improved the proficiency to discriminate motion direction in random-dot-motion tasks in healthy adults. Van der Groen et al. (2019) added visual noise to a visual stimulus in a binocular rivalry experiment and showed clear SR. In another study, participants were asked to extract image features from visual noise and the performance in the task was best at non-zero amounts of external noise (Simonotto et al., 1999). Participants showed an inverted U-shaped function (SR) in the task just like in our lab-based experiment. Similar enhancements in detection and discrimination tasks were also found in the auditory domain where auditory noise was used to enhance hearing (Zeng et al., 2000).

For behaviorally relevant stimuli such as those used in our experiments (letters), Piana et al. (2000) showed that optimal amounts of noise dramatically increased performance in a letter detection task. Itzcovich et al. (2017) also found SR for all of their visually impaired participants when they used a letter-detection-in-visual-noise task. Our finding of SR in a similar task are consistent with these others.

### 6.2. Stochastic resonance and ASD

We investigated the link between autism traits (and by inference ASD) and SR through external noise manipulations. We modeled our data with an extension on the *M*-alternative choice signal detection model, to include a threshold, allowing for SR for sub-threshold stimuli to occur. Fitting the model to our data we found mixed evidence for the hypothesis that suggests increased performance in ASD on selected tasks may be due to SR, mediated by high internal noise in ASD (Simmons et al., 2009).

In support of the hypothesis, our *M*-alternative choice task modeling predicted that higher AQ group (or ASD) shows enhanced performance at low noise levels compared to lower AQ group (or TD), with this increased performance showing a relatively rapid decline with increased noise levels. This was indeed found in Experiment 2, but not in Experiment 1. The model also predicted that the lower AQ group (or TD) would show SR, before showing a decline in performance at high noise levels. This is qualitatively shown in Experiment 2 as well. Although no significant SR was found in Experiment 2 overall, model fitting showed SR in 12 out of 30 individuals. The prediction that internal noise estimates would correlate positively with AQ was not supported by our data, although a positive trend was present in the data.

Overall, our results are the first indirect experimental evidence that SR could indeed be the explanation for improved performance in ASD on some tasks. Note that our results still need to be interpreted with caution, as it is difficult to conclude and extend our findings to the ASD population without performing similar sets of experiments in that population. Moreover, our sample only consisted of a limited range of AQ scores for both experiments. For instance, while we used a median split to compare lower AQ scores with higher AQ scores, most participants with higher AQ scores are well within the neurotypical range of scores. Additionally, it is possible that our estimates of internal noise level could be improved by measuring internal noise directly, for example by using a double-pass psychophysical technique (Vilidaite et al., 2017).

### 6.3. Colored noise

We also explored whether colored noise, such as infrared, brown, and pink noise, could exhibit an SR-like phenomenon. Even though we found significant differences between the different noise types, this difference was mostly confined to high external noise levels. We found no overall significant effect of SR in the data, and this was true for all noise colors. Other research has found some success when investigating SR with colored noise in other domains. For instance, brown noise has resulted in SR in a neuronal model (Coelho et al., 1999). Experiments in rat sensory neurons have also suggested that 1/*f* noise can be better than white noise for improving the response of a neuron to a weak signal under certain circumstances through SR (Nozaki et al., 1999). Some calculations have also suggested that pink noise can show an SR effect and can enhance performance of a system (Hänggi, 2002). Lastly, in a recent paper, SR was experimentally studied in an artificial neuron and the author demonstrated that pink noise enhanced the input signal by up to 20 times more when compared to white noise in neural circuits (Pinto, 2021). Our research is the first to investigate the impact of colored noise in a behavioral task. Although we did not find a statistical difference in the type of noise used, we see that brown noise impaired performance more at high external noise levels. Perhaps, future research can investigate the effects of colored noise in a laboratory-based (and not online) behavioral task and compare the results with our findings.

### 6.4. Letter confusion, and SR

We found SR at accuracy levels much higher than expected from the model that we proposed (where the maximum accuracy is (1 + *M*/2 *M*). When we plotted confusion matrices, we found that the letter stimuli were not equally confusable, resulting in better than chance guesses (and higher accuracy). This is line with past research, which has suggested that some letter pairs or other groups are more confusable, as they share similar features (Townsend, 1971; Gervais et al., 1984). Consequently, some letters are easy to identify, while others are more difficult to identify, depending on the task (Jordan et al., 2003). For example, we found confusions between letters E and F, which is consistent with findings from Townsend (1971). Also, letters such as D, G, O, Q, and U have shown poor performance in letter identification tasks because of confusions among them (Mueller and Weidemann, 2012).

Therefore, having non-confusable letters in the online experiment allowed the participants to guess the letter correctly at zero and high noise levels at levels higher than expected. Instead of guessing 1/9, it appeared that for most letters the guessing was closer to ½, elevating baseline “guess” rates. When incorporating this knowledge into our model, the model fitted the elevated levels of accuracy quite well. At these higher accuracy levels, finding SR is more difficult (as there is less room to ceiling performance), thus the selection of letters can impact performance and the possibility of SR.

### 6.5. Inconsistent results across the two experiments

Given the different pattern of results in the two experiments, it is worth discussing the possible origins of these differences. The two experiments had several key differences in approach. The environment of the experiments was different, with the online condition being less well controlled than the lab-based experiment. Second, the experiments used different set of letters which can impact the appearance of SR.

There were also some more nuanced differences between the experiments. For instance, the letter stimuli, and the noise stimuli were larger in the online experiment. Even though we did not control the distance from the screen where people sat, a standard distance from the screen would result in a stimulus that is much larger in the online experiment. For instance, the stimulus size in the online task would have been close to seven degrees of visual angle, but for the lab-based experiment it would have been around two degrees. Further, the check sizes were smaller for the lab-based experiment compared to the online task (respectively 1px and 3.44px check sizes). Given that the relationship between spatial frequency content and letter sizes influences letter detectability (Alexander et al., 1994), as well as the possible impact of spatial averaging on the smaller check sizes in the lab-based experiment, the different check sizes could possibly explain the difference in results. Check sizes relative to the target letters were comparable (approximately 85 vs. 87 checks per letter).

Other factors such as the participants, the threshold procedures, and the number of trials also differed across the two experiments, which also could have contributed to the difference in results between them. Our online study also did not account for factors such as differences between participants in internet speed and computer performance.

### 6.6. The impact of showing SR in an online setting

Previous studies have shown that SR can be achieved in a well-controlled environment [for example see (Enders et al., 2013; Treviño et al., 2016; Itzcovich et al., 2017)]. In most of these experiments, participants are in an environment where they are adhering strictly to procedures provided by the researchers. For example, in visual detection tasks, these procedures may include sitting at an appropriate distance from the computer, using a chin rest to have an appropriate head position, and finally, using an eye tracker to monitor participants’ eye movements (this is generally done to eliminate trials in the analysis where the participants were distracted). This environment is generally ‘quiet’, which makes it easier for an SR effect to occur when external noise is added to the stimuli. While our results from the lab-based study followed a similar procedure, and found the expected results, the online experiment provided us with some new insights. We found no overall strong SR. However, SR did seem to be present for more difficult-to-identify letters. Also, when our model was adjusted for higher accuracies at lower noise levels, we found that there was some SR for all letters in the overall data for the online experiment. Additionally, 12 out of 30 participants showed SR in the experiment when we fitted models for individual participants. This suggests that SR can occur even in relatively uncontrolled settings, showing a level of robustness that is important if visual SR were to be used to achieve real-life improvements in vision.

In conclusion, our experimental data and modeling provide some insight and support for the hypothesis that higher internal noise in ASD can explain better task performance on some tasks. However, our results were equivocal, as Experiment 1 did not find group differences in SR, while Experiment 2 did.

## Data availability statement

The raw data supporting the conclusions of this article will be made available by the authors, without undue reservation.

## Ethics statement

The studies involving human participants were reviewed and approved by University of Canberra Human Research Ethics Committee. The patients/participants provided their written informed consent to participate in this study.

## Author contributions

PR: data collection, data curation, formal analysis, investigation, software, visualization, and writing—original draft. KM: data collection and writing—original (experiment 1–materials and procedure). LW: software and writing—review and editing. JB: formal analysis, investigation, modeling, software, supervision, and writing—review and editing. All authors contributed to the article and approved the submitted version.

## Funding

This research was funded partially by the Australian Government through the Australian Research Council Discovery Project (project number DP220100406).

## Conflict of interest

The authors declare that the research was conducted in the absence of any commercial or financial relationships that could be construed as a potential conflict of interest.

## Publisher’s note

All claims expressed in this article are solely those of the authors and do not necessarily represent those of their affiliated organizations, or those of the publisher, the editors and the reviewers. Any product that may be evaluated in this article, or claim that may be made by its manufacturer, is not guaranteed or endorsed by the publisher.

## Footnotes

^1^Note that we will be using identity-first language throughout this manuscript as we are aligned with the consensus of using identity-first language as opposed to person-first language (Botha et al., 2023).

^2^When we discuss the model, we add in parentheses that the higher AQ group results could also apply to ASD groups. This is because our higher and lower groups refer to high and low noise, where the high group could be both higher AQ (within the TD population) or alternatively ASD.

^3^ www.gorilla.sc
