# Supplementary material for: Does stochastic resonance improve performance for individuals with higher autism-spectrum quotient?
Source: Front Neurosci. 2023 Apr 14;17:1110714. doi: 10.3389/fnins.2023.1110714 (PMC10140507; doi:10.3389/fnins.2023.1110714)
Supplement: Supplementary file 1 [file Data_Sheet_1.pdf]

Supplementary Material

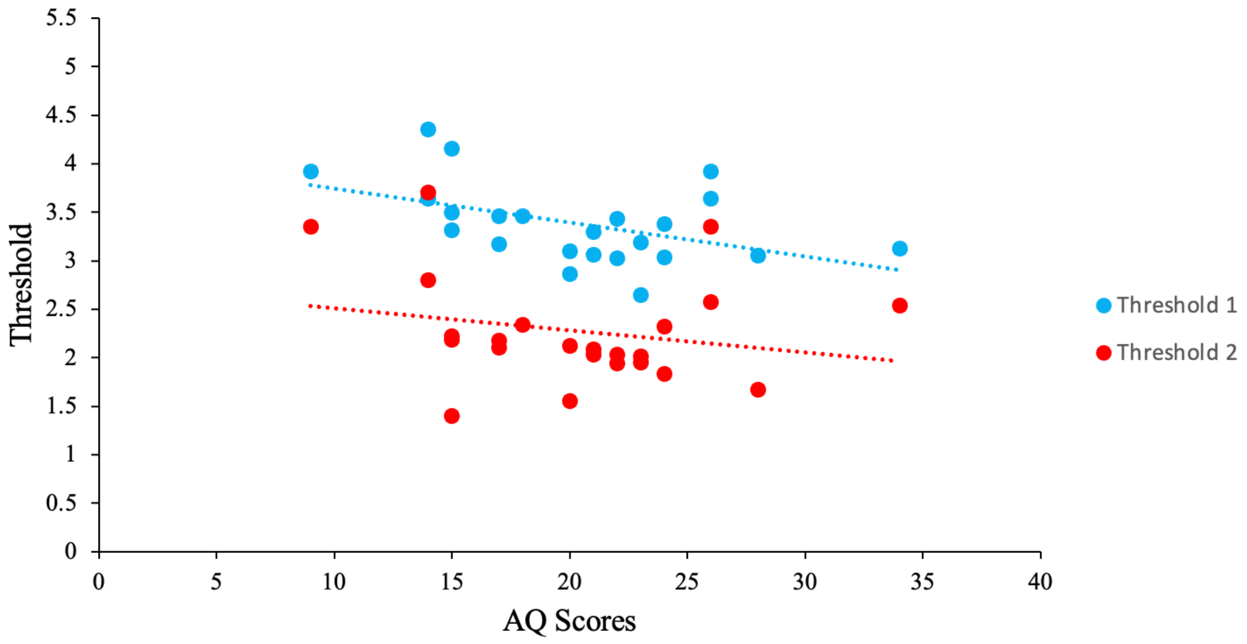

Supplementary Figure 1. Improvement in detection thresholds were not associated with AQ scores in Experiment 1. Lower threshold values indicate better performance.

Supplementary Figure 2

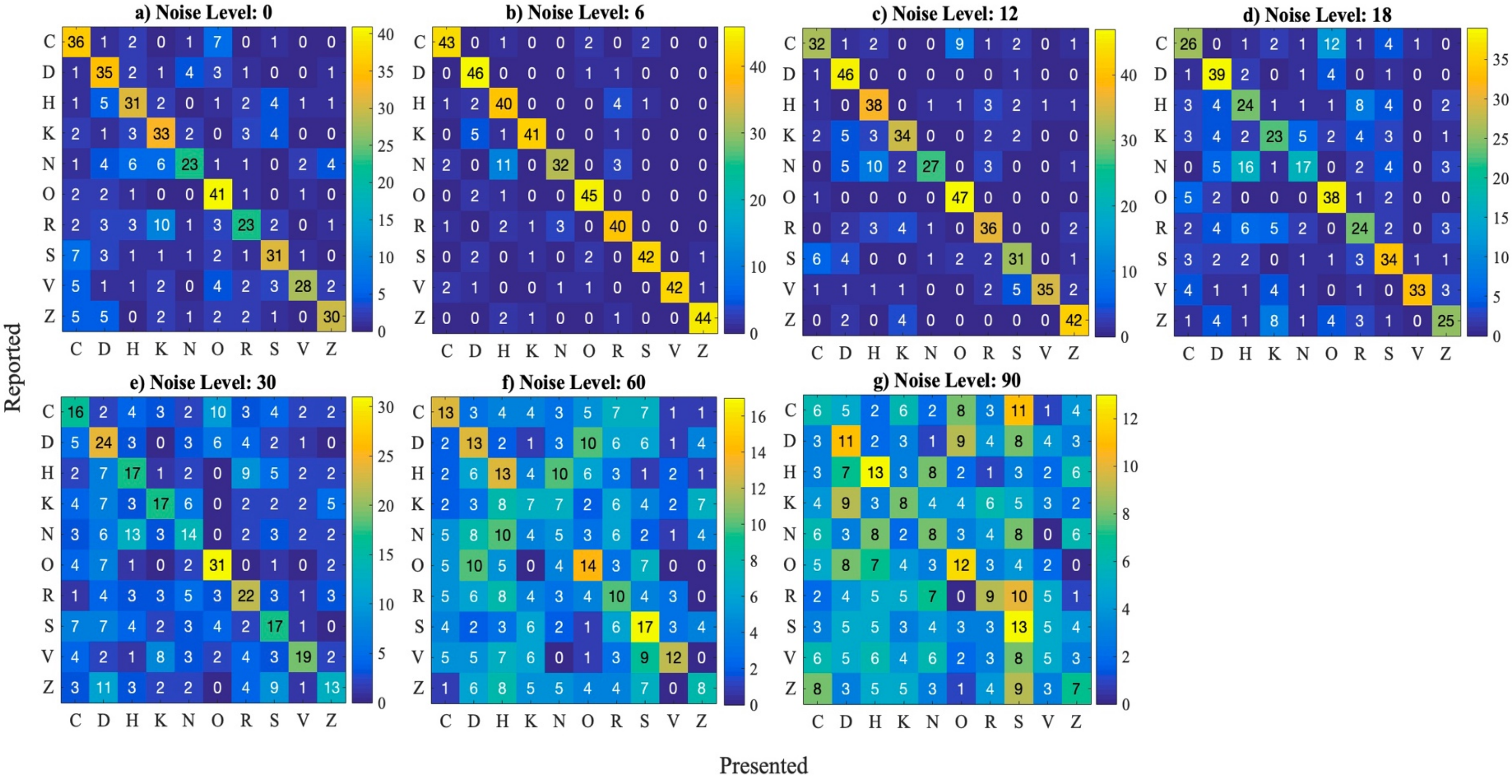

Supplementary Figure 2. Confusion matrix depicting how accurately the letters in the online experiment were correctly guessed across different noise levels in Experiment 1.

Supplementary Figure 3

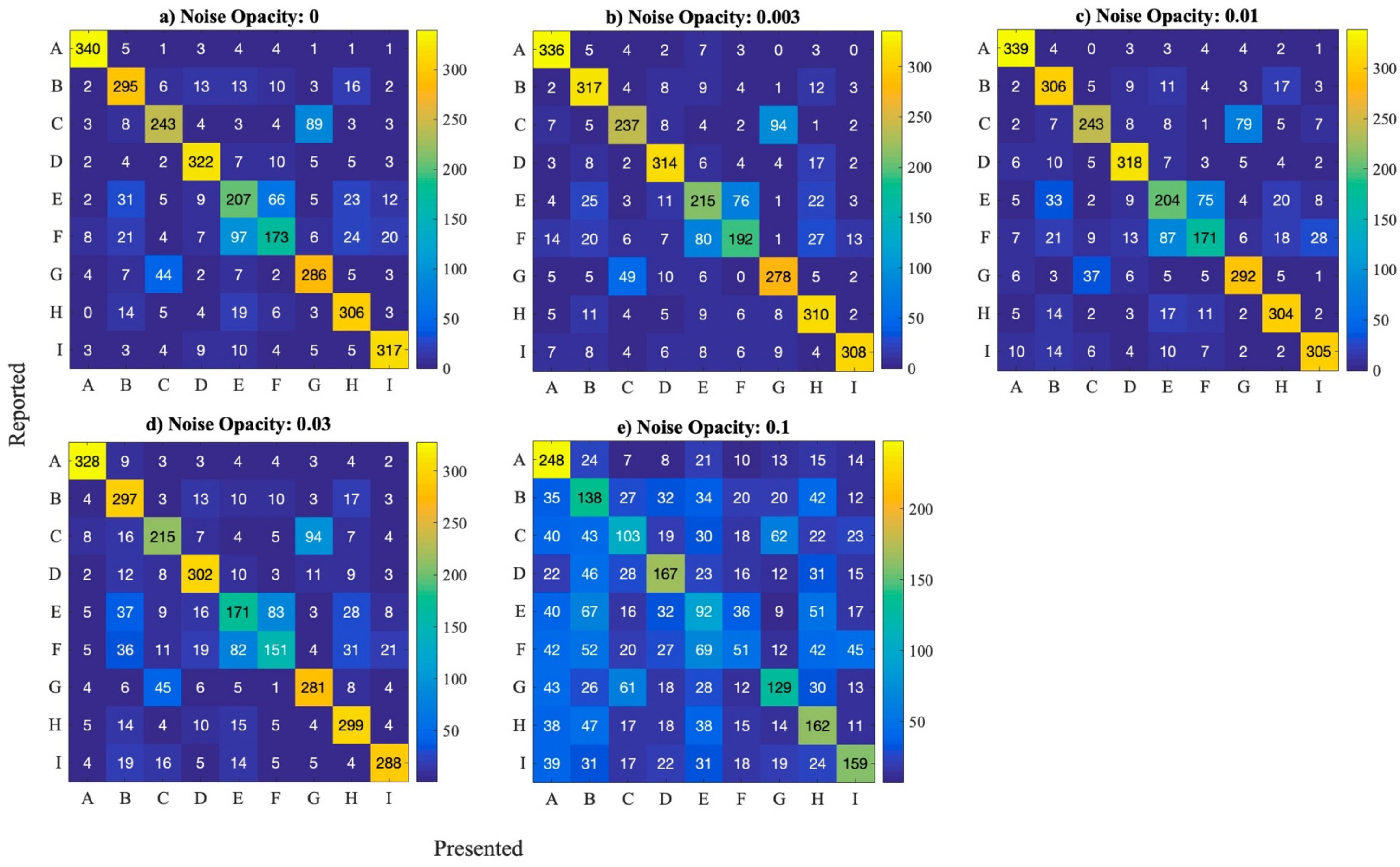

Supplementary Figure 3. Confusion matrix depicting how accurately the letters in the online experiment were correctly guessed across different noise levels in Experiment 2.

Supplementary Figure 4

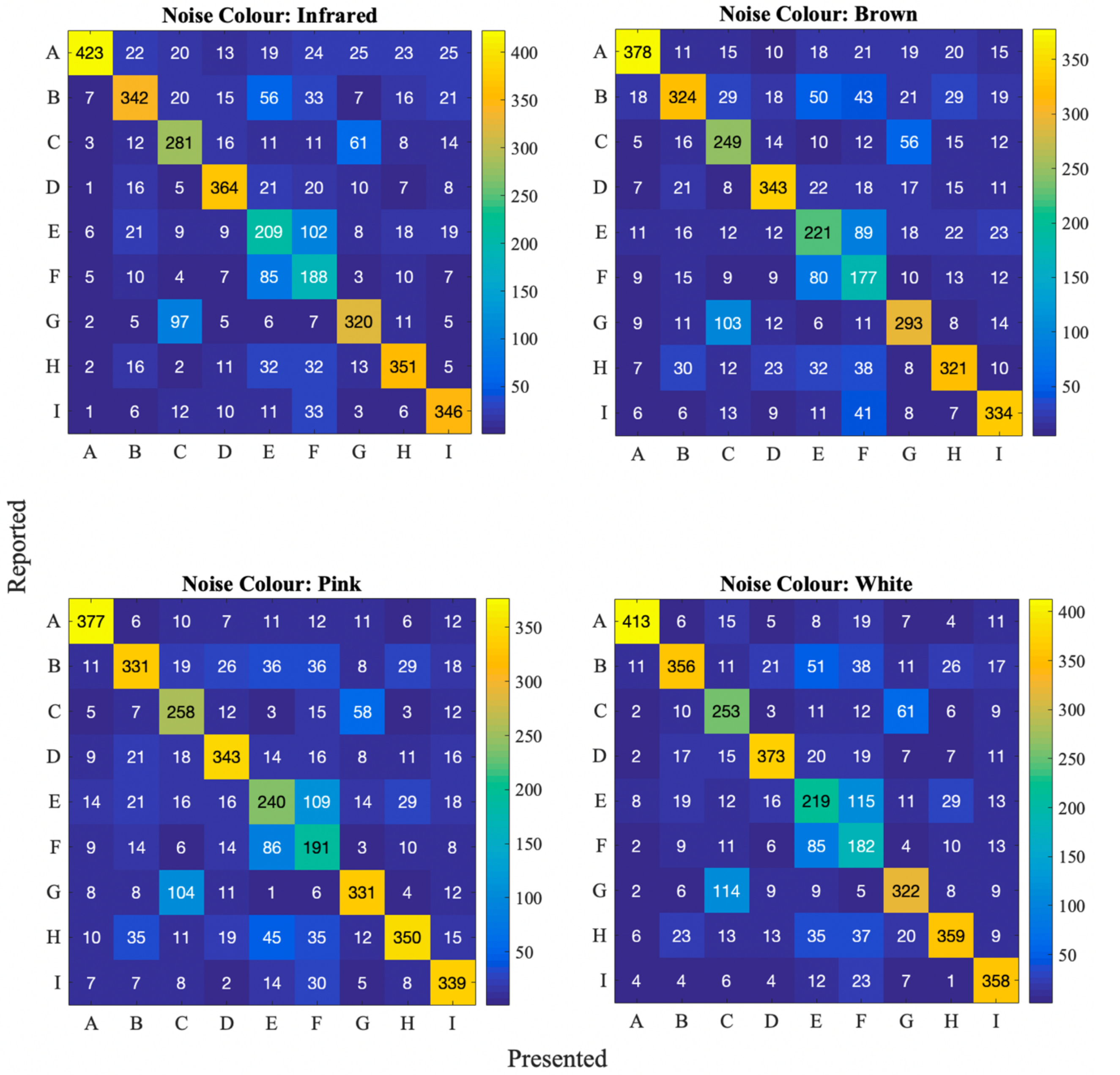

Supplementary Figure 4. Confusion matrix depicting how accurately the letters in the online experiment were correctly guessed across different noise colours in Experiment 2.

Supplementary Table 1

Table 1: Table showing comparisons between different models

Models	Fixed parameters	Fitted parameters	AIC (average)	Error (average)	Number of participants the model fit best (out of 30)
Model 1	M_2	τ, s & internal noise	-46.42	0.96	12
Model 2	M_2	τ (fitted independently for all letters); s & internal noise (combined over all letters)	-40.36	1.06	4
Model 3*	-	M_2, τ, s, & internal noise	-51.59	0.71	14
Model 4	s	M_2, τ, & internal noise	-37.50	0.86	0

* Indicates the best model.
